# Supplementary material for: Dual carbon sequestration with photosynthetic living materials
Source: Nat Commun. 2025 Apr 23;16:3832. doi: 10.1038/s41467-025-58761-y (PMC12019168; doi:10.1038/s41467-025-58761-y)
Supplement: Supplementary file 1 — Supplementary Information [file 41467_2025_58761_MOESM1_ESM.docx]

**Supplementary Information**

**Dual carbon sequestration with photosynthetic living materials**

Dalia Dranseike^1,#^, Yifan Cui^1,#^, Andrea S. Ling^2^, Felix Donat^3^, Stéphane Bernhard^1^, Margherita Bernero^4^, Akhil Areeckal^1^, Marco Lazic^1^, Xiao-Hua Qin^4^, John S. Oakey^5^, Benjamin Dillenburger^2^, André R. Studart^6^, and Mark W. Tibbitt^1,^*

^1^ Macromolecular Engineering Laboratory, Department of Mechanical and Process Engineering, ETH Zurich, Zurich, Switzerland.

^2^ Digital Building Technologies, Institute of Technology and Architecture, ETH Zurich, Zurich, Switzerland.

^3^ Laboratory of Energy Science and Engineering, Department of Mechanical and Process Engineering, ETH Zurich, Zurich, Switzerland.

^4^ Institute for Biomechanics, Department of Health Sciences and Technology, ETH Zurich, Zurich, Switzerland.

^5^ Department of Chemical and Biomedical Engineering, University of Wyoming, Laramie, WY, USA.

^6^ Complex Materials, Department of Materials, ETH Zurich, Zurich, Switzerland.

^#^ equally contributing authors

* corresponding author: [mtibbitt@ethz.ch](mailto:mtibbitt@ethz.ch)

# Supplementary Methods

# Supplementary Method 1: Calculation of dissolved inorganic carbon in BG11*–*ASNIII medium with CO2SYS program

To evaluate the amount of dissolved inorganic carbon in the culture medium, an established CO2SYS program was used for calculating the equilibrium of dissolved inorganic carbon (DIC) at each time point. The CO2SYS program requires a minimum of two inputs under a given temperature and pressure, to calculate other parameters in an equilibrium state. These parameters include, but are not limited to, total alkalinity, total dissolved inorganic carbon, *pH* and either fugacity or partial pressure of CO_2_.

Specifically for our system, we measured the pH and total alkalinity of the collected BG11*–*ASNIII medium every 5 days, with salinity as an additional control parameter. Total alkalinity was obtained through a titration method[^1^](https://sciwheel.com/work/citation?ids=17001610&pre=&suf=&sa=0&dbf=0). In brief, BG11*–*ASNIII medium was collected, and the volume was measured. This collected medium was titrated against a 0.001639N sulfuric acid solution with a constant stirring speed of 100 rpm. *pH* values were recorded until the medium reached a *pH* value of below 3.5. The medium alkalinity was calculated by using the Alkalinity calculator from USGS website with the inflection point method (https://or.water.usgs.gov/alk/). Salinity was measured indirectly by measuring the conductivity of the collected culture media. Conductivity of the solution was measured using Dynamic Light Scattering Analyzer (Anton Paar, LITESIZER™ 500). The solutions were placed into an omega cuvette (Anton Paar) and measured at 25 °C with 20 scans. The properties of each sample were measured three times reported as a conductivity average. Conductivity of the medium was then calculated based on the correlation shown in Equation S1[^2^](https://sciwheel.com/work/citation?ids=17001599&pre=&suf=&sa=0&dbf=0).

*S*=0.4665*x*^1.0878^ (r^2^ = 0.98799) Equation S1

*pH* value, total alkalinity and salinity for each time point were used as the input values for the established python (version 3.10)-based calculation of CO2SYS (version 1.8.3.4). The amount of total borate was set to be 0.046 µmol kg^-1^ which is the amount of borate added in the BG11–ASNIII medium. The total amount of dissolved inorganic carbon was calculated by the program below.

import pandas as pd

import PyCO2SYS as pyco2

# Perform CO2 calculations

# par1_type=3: pH; parl2_type = 1: total alkalinity

results = pyco2.sys(par1=6.263, par1_type=3, par2 =472, par2_type=1, total_borate=0.046, salinity=31.27)

# Create a DataFrame from the results

df = pd.DataFrame.from_dict(results, orient='index', columns=['Values'])

# Specify the file path to save the Excel file

file_path = 'abioday30.xlsx'

# Write the DataFrame to Excel

df.to_excel(file_path, index=True)  # index=True to include the DataFrame index in the Excel file

print("Excel file written successfully.")

# Supplementary Method 2: Equivalent CO_2_ sequestration calculation in living materials

To evaluate the mass of CO_2_ converted into biomass and inorganic precipitates within hydrogel matrix we weighed the dry mass of randomly selected abiotic and biotic samples over the incubation period of 30 days.

${mass}_{abiotic}={mass}_{F127}+{mass}_{F127-BUM}$ Equation S2

${mass}_{biotic}={mass}_{F127}+{mass}_{F127-BUM}+{mass}_{biomass}{+mass}_{precipitates}$ Equation S3

As the hydrogel is composed of 13.2 wt% Pluronic F127 and 7.3 wt% photo-cross-linkable F127-BUM, the non-cross-linked F127 diffused out over time and the dry mass of abiotic samples decreased to 45 ± 7% of the original mass (Supplementary Fig. 16a). The same effect was expected for biotic samples. However, the final mass after 30 days of incubation was 81 ± 9% (Δ(*Mass_biotic_* – *Mass_abiotic_*)) of the initial one as the reduction of polymer content was partially replaced by the generated biomass and inorganic precipitates.

To quantify the extent of insoluble precipitates, thermal decomposition was performed to remove the organic biomass and polymer matrix. Dry abiotic or biotic samples (*n_samples_* = 5 and *n_replicates_* = 5) with a total mass of 10-20 mg per replicate were placed in a crucible for thermal decomposition at 600 °C. The biotic sample mass after thermal decomposition, which corresponded to the mass of the insoluble carbonate precipitates, was 5 ± 2% (Supplementary Fig. 16b). The remaining mass of the abiotic samples was 1 ± 1% and, therefore, we considered it insignificant.

To convert this into the amount of sequestered CO_2_ we used:

*V*_sample_ = 40 µL – from the design of disc samples

*m*_dry sample_ = 4 mg – from the measured average of biotic samples on day 30

*m*_inorganic precipitates per sample_ = 0.2 mg – as 5% of sample mass based on the measured mass after thermal decomposition

number of samples per 1 mL of hydrogel = 1000 µL (0.04 µL)^-1^ = 25

MW(CaCO_3_) = 100 g mol^-1^

MW(MgCO_3_) = 84 g mol^-1^

In case of a mixture of 70% CaCO_3_ and 30% MgCO_3_, as suggested by the XRD results in the calcite peak shift[^3^](https://sciwheel.com/work/citation?ids=15074189&pre=&suf=&sa=0&dbf=0), average MW= 95.2 g mol^-1^,

Then, the number of mole of CaCO_3_ precipitates per 1 mL of hydrogel:

$n (precipitates)=\frac{m_{inorganic precipitates per sample}}{M_{w}(\mathrm{CaCO}_{3})}\cdot25$ Equation S4

This results in approximately 50 µmol of CO_2_ sequestered as inorganic precipitates per 1 mL of living material or 2.2 ± 0.9 mg per 1 mL of living material when considering the precipitates to be pure CaCO_3_. Slightly higher value of 52.5 µmol of CO_2_ sequestered per 1 mL is obtained when considering a mixture of 70% CaCO_3_ and 30% MgCO_3_.

# Supplementary Method 3: Direct CO_2_ measurement in a closed environment

A closed CO_2_ measurement system was built with a 250 mL airtight container, a CO_2_ sensor (Sensirion I2C SCD4x Multiple Function Sensor), and an Arduino microcontroller board (Uno r4 minima). An SD card reader was connected to the Arduino board to record the data. Two 8.2 kΩ resistors were added into the electric circle of the sensor to amplify the voltage signal of the two data cables. A 0.25 mL sample with approximately 10 mL of culture medium was placed in each measurement container. The containers were opened every day and were allowed to equilibrate with atmospheric air to replenish the CO_2_ within the closed system. The CO_2_ concentrations were recorded on the SD card every 4–5 s.

# Supplementary Method 4: Bacteria culturing conditions

Culture medium conditions in a mixture of BG11 and ASNIII medium were adapted from the guidelines of the supplier. ASNIII medium was prepared by mixing all components (Supplementary Tables 4 and 5) in deionized water, then autoclaved and supplemented with A5+ Co Trace metals (1x from initial 1000x stock solution) and vitamin B_12_ (0.010 g L^-1^). ASNIII medium was used at 2x final concentration. BG11 medium was used as received (100x stock solution) at a 2x concentration.

The bacteria culture was cultivated in Erlenmeyer flasks on a shaker plate (150 rpm) at 30 °C and illumination of approximately 180 μmol photons m^-2^ s^-1^ using 12-hour day (on)/night (off) cycle.

# Supplementary Method 5: F127-bis-urethane methacrylate synthesis

F127-bis-urethane methacrylate (F127-BUM) was synthesized by adapting the previously reported method by Millik et al. [^4^](https://sciwheel.com/work/citation?ids=10883750&pre=&suf=&sa=0&dbf=0). Pluronic F127 (*m* = 30 g) was vacuum dried for 2 h and then dissolved in anhydrous DCM (*V* = 275 mL) by magnetic stirring at 30 °C until Pluronic F127 was fully dissolved. 6 drops of dibutyltin dilaurate was then added. A mixture of 2-isocyanatoethyl methacrylate (*V* = 1.75 mL) and DCM (*V* = 25 mL) was added to the Pluronic F127 solution at a rate of 1 drop per second and the addition process was allowed to proceed under argon atmosphere at 30 °C overnight.

The reaction was then quenched with methanol (*V* = 30 mL), concentrated by a rotatory evaporator at 45 °C, and subsequently precipitated in 800 mL of ice-cold diethyl ether. The reaction product was recovered by centrifugation (at 3000 g for 10 min at 4 °C) followed by two additional washing with diethyl ether and centrifugation steps. Synthesized F127-BUM was then dried under vacuum and stored at -20 °C until use. Pluronic F127 modification was confirmed via ^1^H NMR (400 MHz, CDCl3): δ 6.06 (m, 2 H), 5.54 (m, 2 H), 5.15 (m, 2 H), 4.16 (m, 8 H), 3.59 (m, 770 H), 3.49 (m, 117 H), 3.35 (m, 56 H), 1.89 (s, 6z H), 1.08 (m, 170 H), (Supplementary Fig. 31).

# Supplementary Method 6: LAP synthesis

Photoinitiator lithium phenyl-2,4,6-trimethylbenzoylphosphinate (LAP) synthesis was adapted from previously reported method [^5^](https://sciwheel.com/work/citation?ids=13097264&pre=&suf=&sa=0&dbf=0)^,^[^6^](https://sciwheel.com/work/citation?ids=1528628&pre=&suf=&sa=0&dbf=0). 2,4,6-trimethylbenzoyl chloride (*V* = 2.9 mL) was slowly added to dimethyl phenylphosphonite (*V* = 2.8 mL) under argon. The mixture was stirred overnight at 25°C. Lithium bromide (*m* = 6.1 g) was dissolved in 2-butanone (*V* = 100 mL) and added to the reaction flask. The temperature of the mixture was then increased to 50 °C for 10 mins to facilitate precipitate formation. The precipitates were recovered using filtration and washed 3 times with 2-butanone. LAP was then dried under vacuum and stored at -20 °C until use. Product formation was confirmed via ^1^H NMR (400 MHz, D2O): δ 7.75 (m, 2 H), 7.60 (m, 1 H), 7.51 (m, 2 H), 6.93 (s, 2 H), 2.28 (s, 3 H), 2.06 (s, 6 H) (Supplementary Fig. 32).

# Supplementary Method 7: Biotic bioink OD_730_ calculation

In order to achieve an equivalent optical density of 0.8 at OD_730nm_ in the bioink, the OD_730nm_ of cyanobacteria suspension prior to encapsulation (OD_730nm,culture_) was measured using UV-visible light spectrophotometer (Lambda 35, Perkin Elmer). OD_730nm_ of pure culturing medium (OD_730nm,medium_) was used as a blank. For every 1 mL of bioink required, the volume of initial cell culture required was calculated as described in Equation S5**.**

$\boldsymbol{V}_{\boldsymbol{cell} \boldsymbol{culture}}=\frac{\boldsymbol{OD}_{\mathbf{730nm},\boldsymbol{culture}}-\boldsymbol{OD}_{\mathbf{730nm}, \boldsymbol{media}}}{\mathbf{0}.\mathbf{8}} \times1 mL$ Equation S5

The growth curve of cyanobacteria culture in BG11*–*ASNIII medium was obtained by measuring the optical density at 730 nm over a period of 36 days and the data is plotted in Supplementary Fig. 35**.** Absorbance of pure culturing medium was also measured as a background reference.

# Supplementary Method 8: Bioink printability characterization

Bioink printability was characterized using strain-controlled shear rheometer (MCR 502; Anton-Paar). The samples were loaded on a temperature-controlled Peltier plate and measured using a 20 mm plate-plate geometry with 0.8 mm gap size at 25 °C. The self-healing properties of the Pluronic F127 based inks were evaluated using dynamic oscillatory time sweep tests with alternating low strain (*ω* = 10 rad s^-1^; *γ* = 0.3%, for *t* = 120 s) and high strain (*ω* = 10 rad s^-1^; *γ* = 1000%, for *t* = 240 s) (Supplementary Fig. 8). The recovery of storage modulus *G′* was evaluated by calculating the ratio between initial *G′* value and that of *G′* after plateau was reached in a low strain interval.

Rotational shear rate measurements were performed in the shear rate range of d*γ*/d*t* = 0.1-100 s^-1^. Shear-thinning index, *n*, and consistency index, *K* of the bioink were obtained by fitting the viscosity dependance on shear rate to Power-law model: 𝜂 = 𝐾 (d*γ*/d*t*)*^n^*^-1^ (Supplementary Fig. 9, Supplementary Table 1).

# Supplementary Method 9: Light dosage calibration for volumetric printer

Light dose testing was performed as per the manufacturer’s instructions. 1 mL of bioink at 4 *°*C was loaded into a calibration quartz glass cuvette (Thorlabs, CV10Q14). Light dosage varying from 360 to 40 mW cm^-2^ was applied at different locations of the cuvette to examine the polymerization efficiency. A second cuvette was then loaded, and the same calibration process was done with a narrower range of light dosages. The final optimal light dosage was calculated by an Apparite software (Readily3D, Switzerland).

# Supplementary Method 10: XRD pattern baseline removal for analysis

The obtained XRD data was imported into Origin 2019 (OriginLab Corporation) for baseline removal. To define the baseline, 40 user-defined points with 2^nd^ derivative anchoring points method were chosen to match the baseline of the plotted 2*θ* against intensity curve. The defined baseline was subtracted from the original intensity values and the curve was plotted again with the baseline subtracted.

# Supplementary Method 11: Sample lyophilization

Cyanobacteria-laden (biotic) samples and abiotic samples were first sterilized in 70% ethanol for 1 h. Ethanol was then discarded and 100 mL of Milli-Q water was added to each sample for 10 min to wash any remaining salt deposited on the surface of the samples from the culture medium. After washing, the samples were frozen (*T* = -20 °C) overnight with fully immersed in Milli-Q water.

A Lyovapor L-300 lyophilizer (BUCHI Labortechnik AG, Flawil, Switzerland) was used with a set pressure of 0.150 mbar and an ice condenser temperature of approximately -101 °C to freeze dry the samples.

# Supplementary Method 12: List of materials

Pluronic F127 (P2443); anhydrous dichloromethane (DCM anhydrous, 900633); dibutyltin dilaurate (C_32_H_64_O_4_Sn, 291234); 2-isocyanatoethyl methacrylate (C_7_H_9_NO_3_, 477060); sodium chloride (NaCl, 71380); magnesium sulfate anhydrous (MgSO_4_, M7506); sodium nitrate (NaNO_3_, 71755); magnesium chloride hexahydrate (MgCl_2_ · 6H_2_O, 63068); citric acid (C₆H₈O₇, C0759); sodium carbonate (Na_2_CO_3_, 13418); ferric ammonium citrate (C_6_H_5+4y_Fe_x_N_y_O_7_ , F5897); BG11 broth (73816); A5+ Co Trace metals (92949); vitamin B_12_ (V2876); EDTA disodium magnesium ((NaOOCCH_2_)_2_NCH_2_CH_2_N(CH_2_COO)_2_Mg · xH_2_O, 317810); alizarin red S (C_14_H_7_NaO_7_S, A5533); lithium bromide (LiBr, 213225), dimethyl phenylphosphonite (C_6_H_5_P(OCH_3_)_2_, 149470); diethyl ether (32203); 2,4,6-trimethylbenzoyl chloride (682519-5G); 2-butanone (C_2_H_5_COCH_3_, 360473) were purchased from Sigma-Aldrich (Germany). Potassium chloride (KCl, 26764.232); methanol (CH_3_OH, 02000347); dipotassium phosphate anhydrous (K_2_HPO_4_ anhydrous, 26931.263); SYTOX Blue dead cell staining (S311348) were purchased from Thermo Fisher Scientific (Switzerland). Anhydrous calcium chloride (CaCl_2_ anhydrous, 349615000) was purchased from Acros Organics. Isopropyl alcohol (≥99.7%, AnalaR NORMAPUR, 67-63-0) was purchased from VWR.

# Supplementary Figures


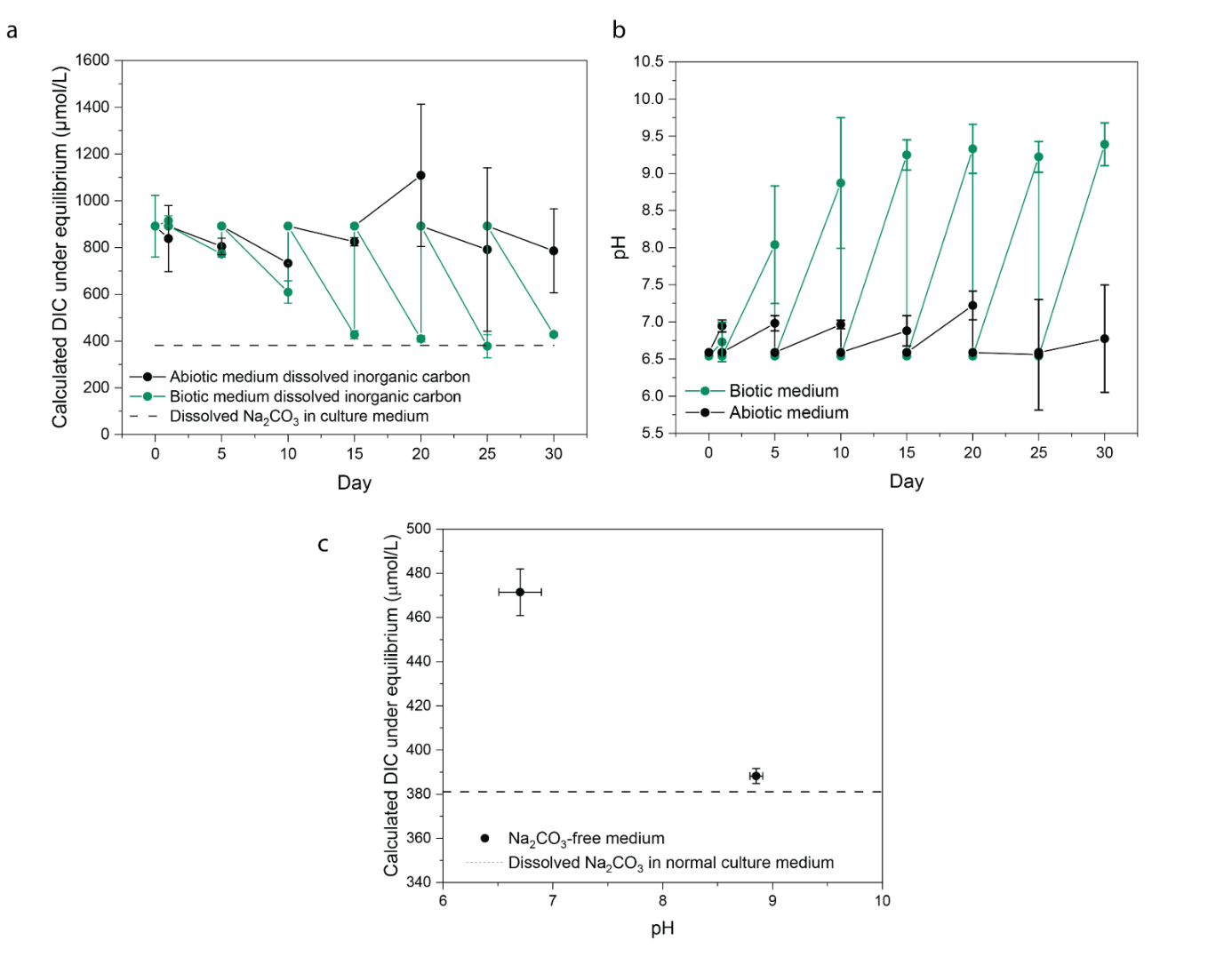


# Supplementary Figure 1. Dynamics of dissolved inorganic carbon (DIC) in biotic and abiotic medium. a) Total amount of dissolved inorganic carbon (DIC) under equilibrium in the re-collected abiotic (black) and biotic (green) medium after every 5-day culture period (*n* = 2 batches, with pooling of *n* = 20 individual samples in each case, biological replicates). The gray dashed line indicates the amount of DIC in the form of Na_2_CO_3_ that was used in simulated seawater medium (BG11–ASNIII). b) pH changes of pooled biotic (green) and abiotic (black) medium every 5 days (n = 3 samples, biological replicates). c) DIC analysis on BG11–ASNIII medium without the addition of inorganic carbon through sodium carbonate (Na_2_CO_3_) at *pH* = 6.7 and *pH* = 8.9 (*n* = 2 samples, biological replicates, black). The gray dashed line indicates the amount of DIC in the form of Na_2_CO_3_ that was used in simulated seawater medium (BG11–ASNIII). Source data are provided in the Source Data file.


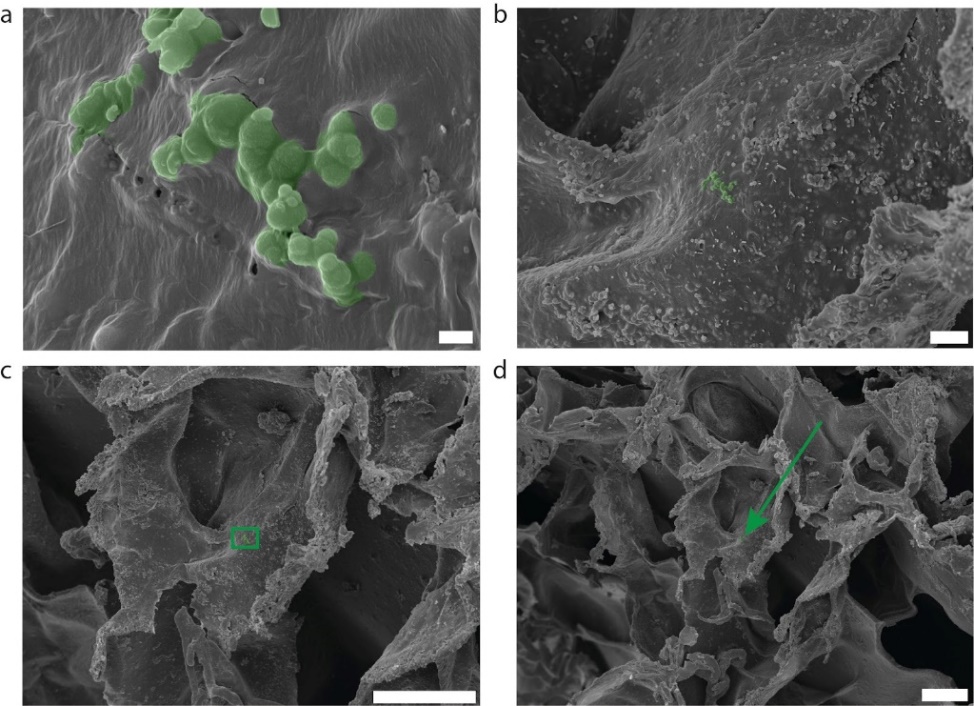


# Supplementary Figure 2. SEM images of the biotic sample at different magnifications. A representative cluster of embedded cyanobacteria *Synechococcus* sp. PCC 7002 are highlighted in green false color to indicate the location within the supporting matrix. It should be noted that the sample was homogeneously populated with embedded cyanobacteria that have not been false colored, as best seen in panel b). Representative scanning electron microscope (SEM) images from *n* = 3 independent experiments with similar results. Scale bars, a) 1 µm, b) 10 µm, c) & d) 100 µm.

*
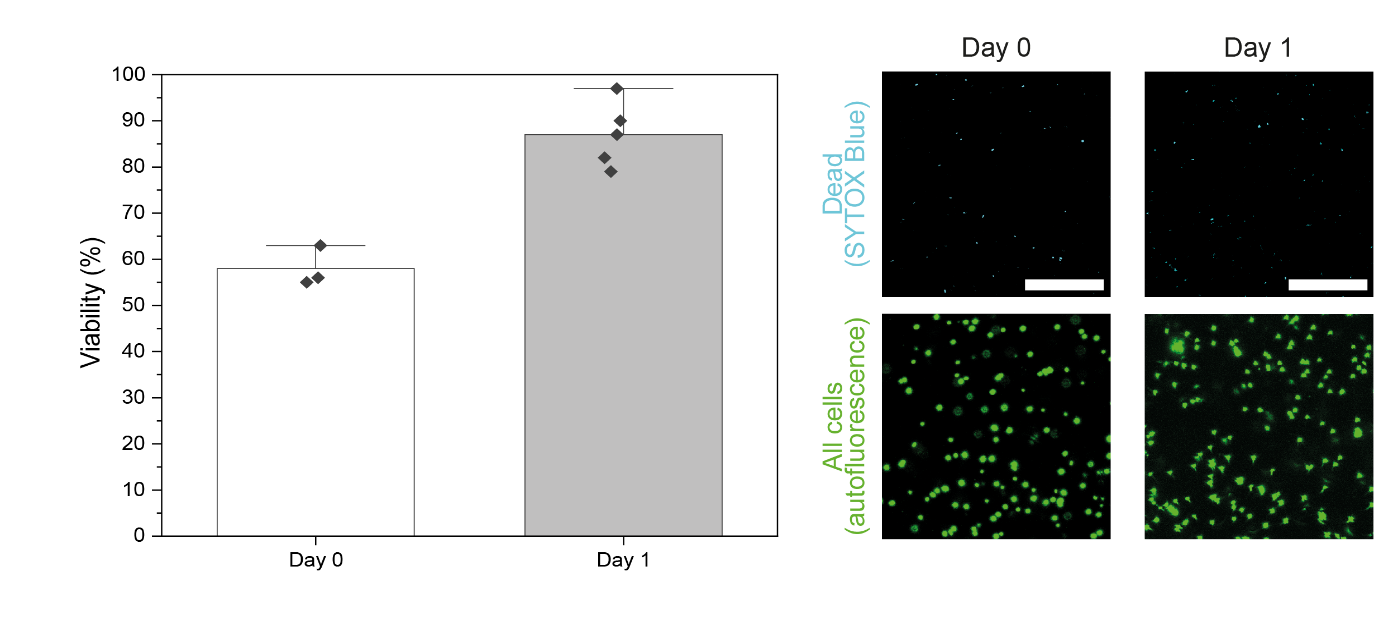
*

# Supplementary Figure 3. Post-printing cell viability. a) Cell viability on day 0 and day 1 after bioprinting. b) Representative maximum intensity Z-projection images of dead cell staining (cyan) and cell autofluorescence (green) (*n* = 3). Scale bar, 100 μm. Source data are provided in the Source Data file.

# Supplementary Figure 4. Transmittance of abiotic F127 based hydrogel with 0.1 wt% Lithium phenyl-2,4,6-trimethylbenzoylphosphinate (LAP) photoinitiator. Cross-linking duration *t* = 2 min. Source data are provided in the Source Data file.

# Supplementary Figure 5. Transmittance of biotic F127 based hydrogel with 0.1 wt% Lithium phenyl-2,4,6-trimethylbenzoylphosphinate (LAP) photoinitiator and PCC 7002 concentration equivalent to OD_730nm_ = 0.8. Cross-linking duration *t* = 1 min. Source data are provided in the Source Data file.


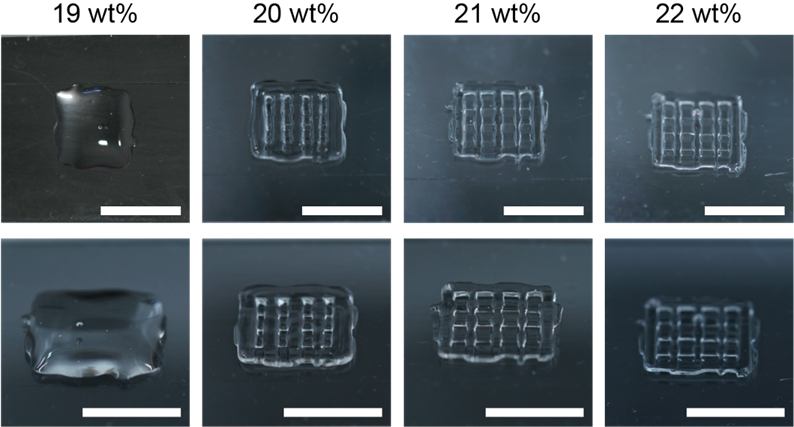


# Supplementary Figure 6. Printability of Pluronic F127 hydrogels of different polymer contents (22G nozzle, printing speed *v* = 5–15 mm s^-1^, printing pressure *P* = 16–45 kPa). Top and planar view. Scale bar, 10 mm. Pictures of the printed samples were taken using a camera (alpha 7R, Sony) equipped with a macro lens (FE 2.8/90 MACRO G OSS, Sony) with consistent lighting.


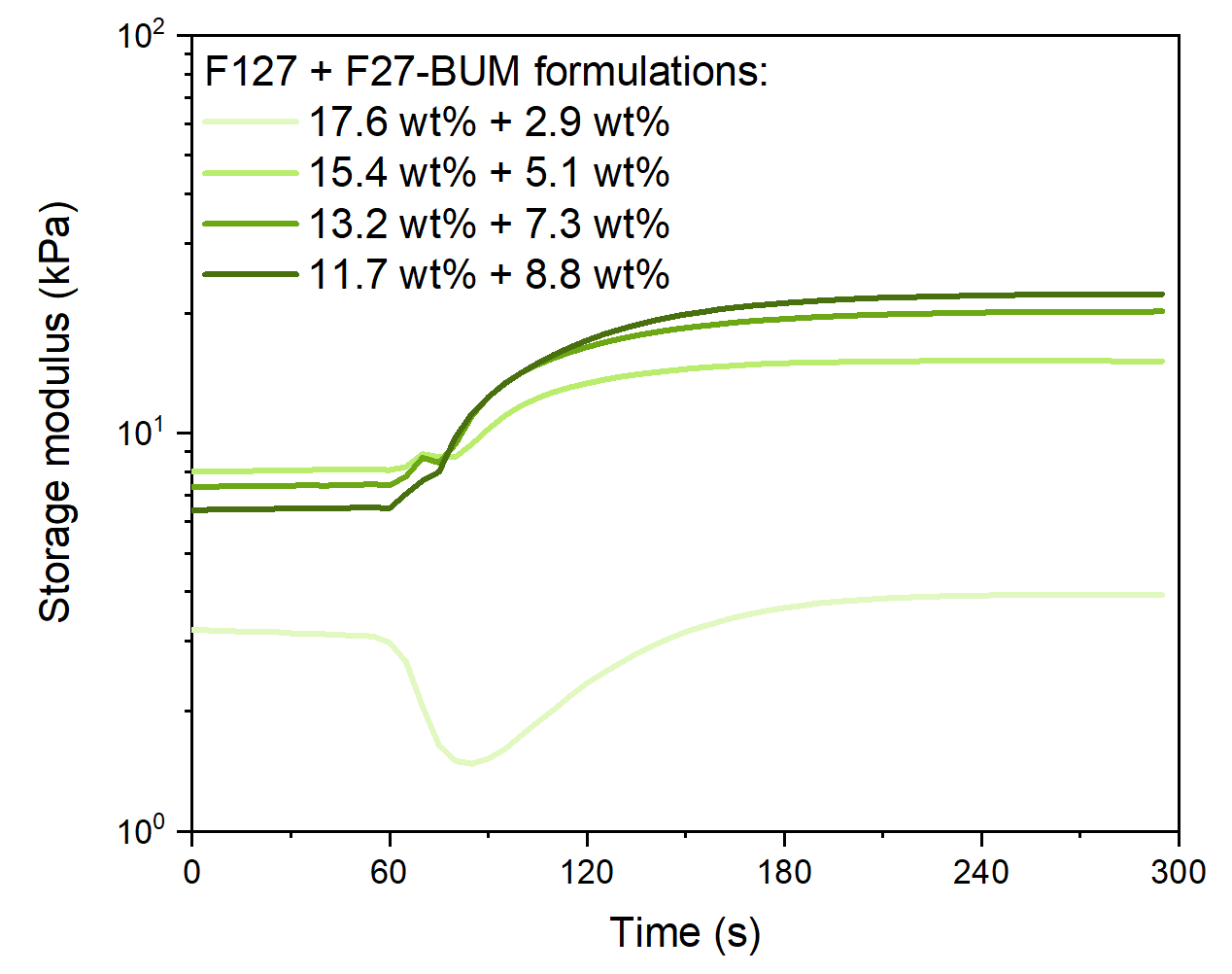


# Supplementary Figure 7. Photo-cross-linking of different ratios of F127 and F127-BUM studied as an increase in storage modulus during the reaction. Representative graphs of different formulations (*n* = 2). The 405 nm light (intensity *I* = 8 mW cm^-2^) was turned on 60 s after the beginning of the measurement. Source data are provided in the Source Data file.

# Supplementary Figure 8. Step strain measurements for Pluronic F127 with alternating intervals at low (shear strain *γ* = 0.3%, angular frequency *ω* = 10 rad s^−1^) and high (*γ* = 1000%, *ω* = 10 rad s^−1^) shear strain amplitude. Representative graph (*n* = 3). F127 exhibited rapid and reproducible recovery of solid-like properties (storage modulus G′ > loss modulus G′′; elastic recovery). Source data are provided in the Source Data file.

# **Supplementary Figure 9.** Shear rate ramp (shear rate d*γ*/d*t* = 0.1–100 s^−1^) for Pluronic F127. Representative graph (*n* = 3). F127 demonstrated a decrease in viscosity with increasing shear rate (shear-thinning). Source data are provided in the Source Data file.


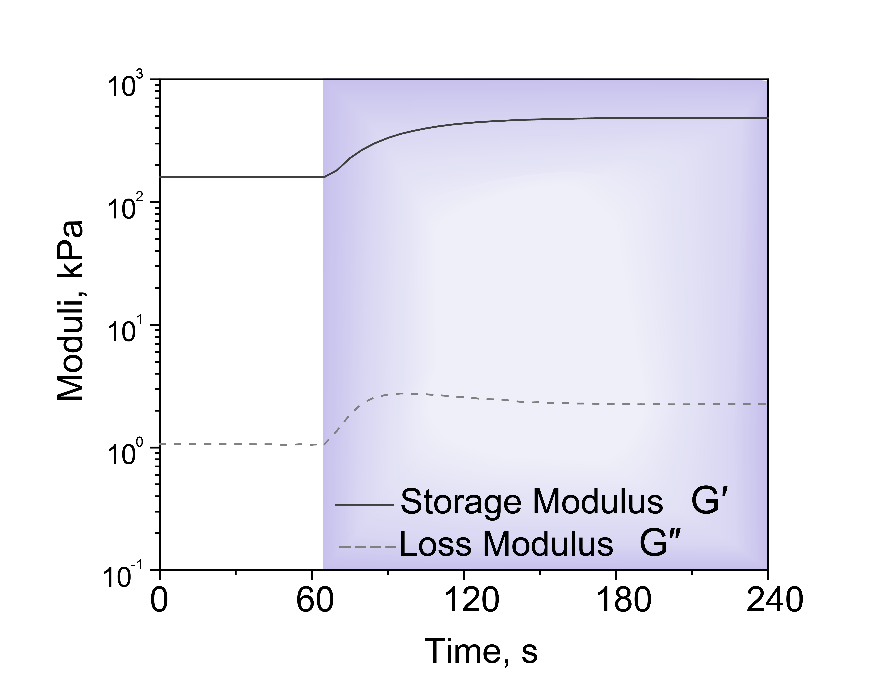


# Supplementary Figure 10. Photo-cross-linking of 13.2 wt% F127 and 7.3 wt% F127-BUM and 0.1 wt% lithium phenyl-2,4,6-trimethylbenzoylphosphinate (LAP) hydrogel using 405 nm (intensity *I* = 8 mW cm^-2^) light (light on in the region marked in violet). Representative graph (*n* = 4). Source data are provided in the Source Data file.

*
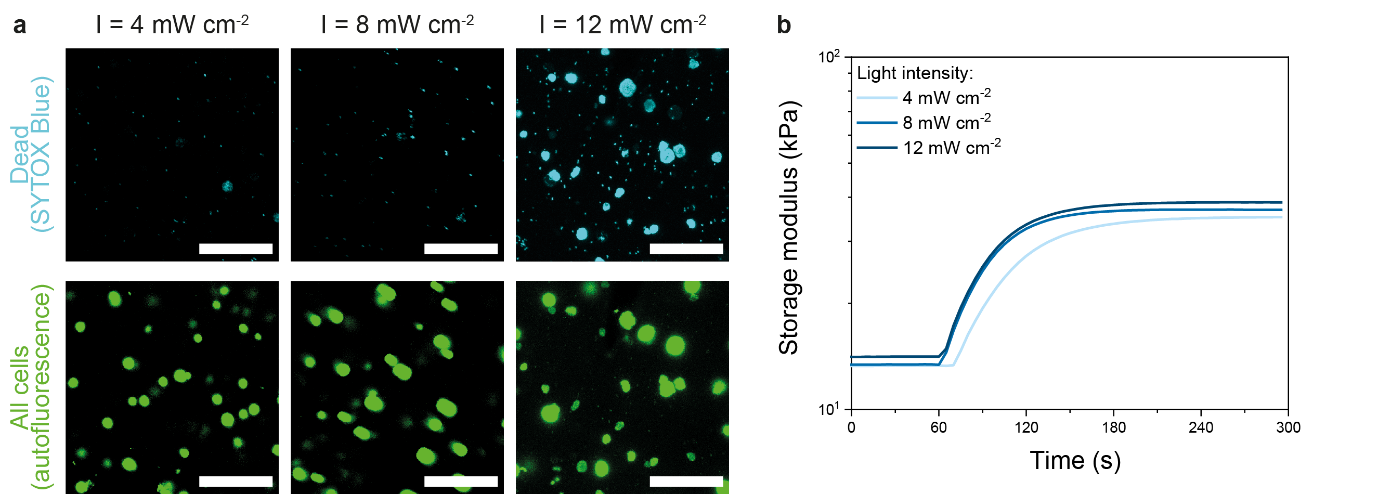
*

# Supplementary Figure 11. Light intensity influence on cell viability. a) Cell viability on day 5 after photo-cross-linking the bioink at different light intensities (SYTOX Blue dead cell staining, cyan; cell autofluorescence, green; maximum intensity Z-projection). Scale bar, 100 μm. b) Bioink photo-cross-linking at different light intensities of 4, 8, and 12 mW cm^-2^ (wavelength *λ* = 405 nm; light turned on at time *t* = 60 s, *n* = 2). Source data are provided in the Source Data file.


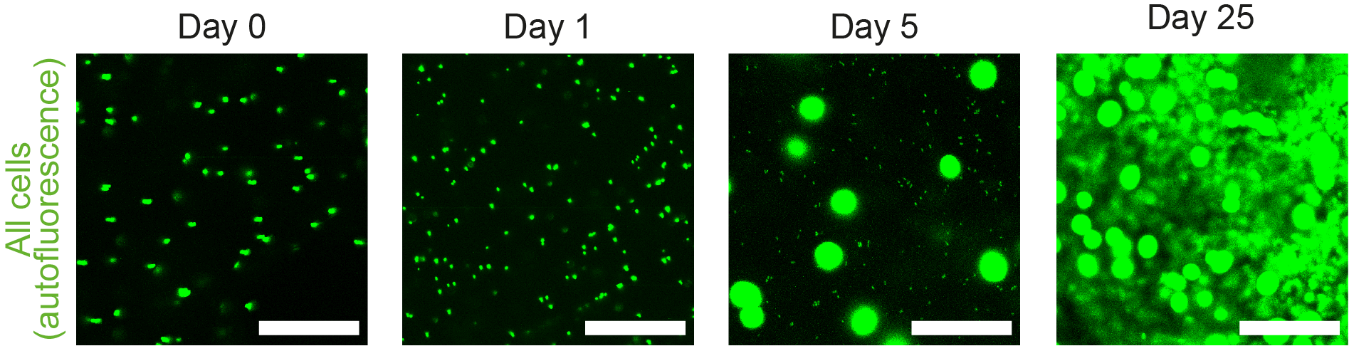


# Supplementary Figure 12. Representative sample maximum intensity Z-projection images at different time points (cell autofluorescence, green), representative images from *n* = 3 independent experiments with similar results. Scale bar, 100 μm.

**a**


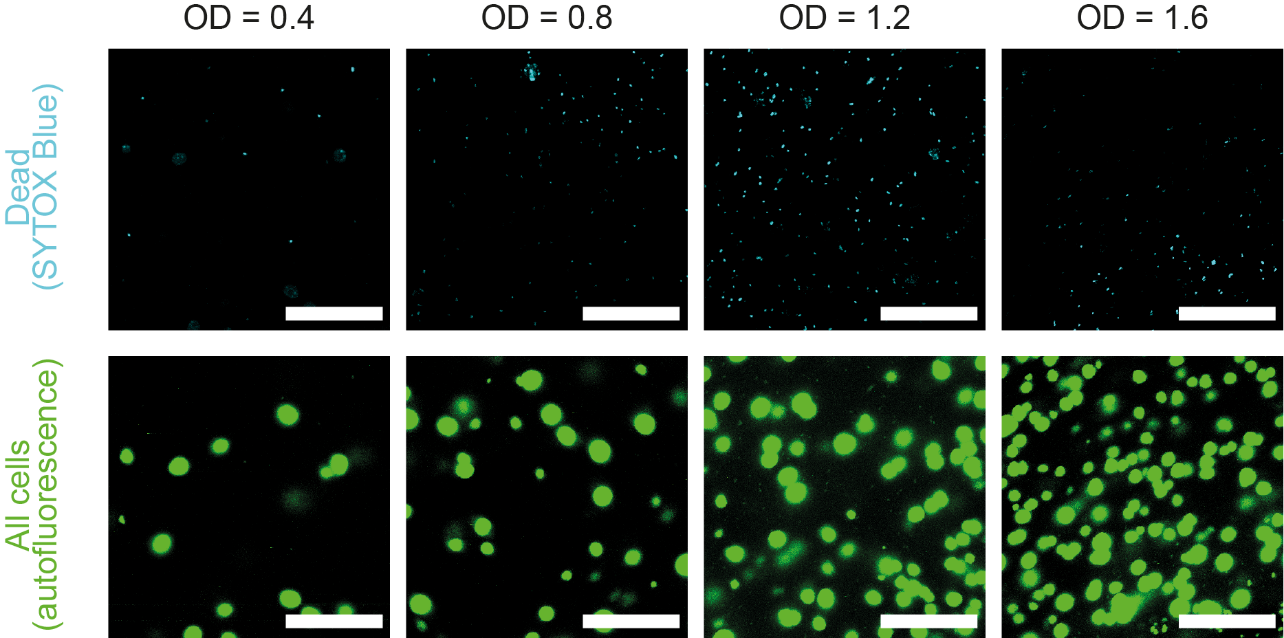


**b**


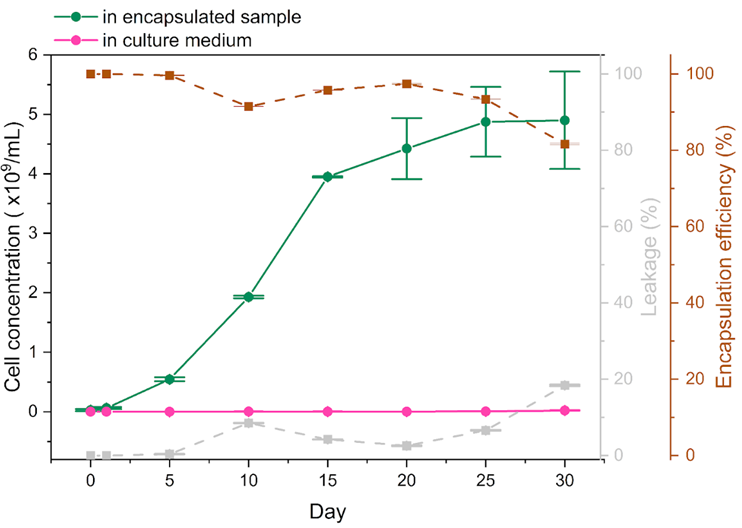


# Supplementary Figure 13. Cell growth and encapsulation efficiency in the living material. a) Cell viability of samples with different starting cell densities (optical density OD in the range from 0.4 to 1.6) on day 5 (SYTOX Blue dead cell staining, cyan; cell autofluorescence, green; maximum intensity Z-projection). Representative images from *n* = 3 independent samples with similar results Scale bar, 100 μm. b) Cell count in the printed disc sample quantified with chlorophyll extraction (green) and cell count in the surrounding culture medium (pink) measured via Coulter Counter (*n* = 3 samples, biological replicates). The right axis indicates the percentage of cells that leaked into the surrounding culture medium normalized to the total number of cells in the printed disc and the culture medium combined (gray), as well as the encapsulation efficiency calculated as 100% – leakage % (brown). Source data are provided in the Source Data file.


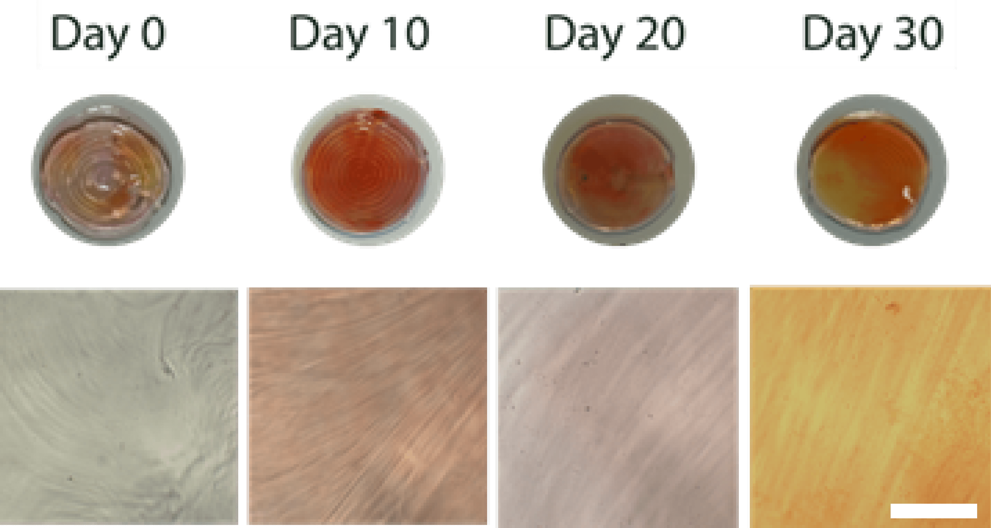


# Supplementary Figure 14. Optical and microscopic images of Alizarin Red S staining on abiotic samples. Representative micrograph from *n* = 5 independent experiments with similar results. Scale bar, 100μm.


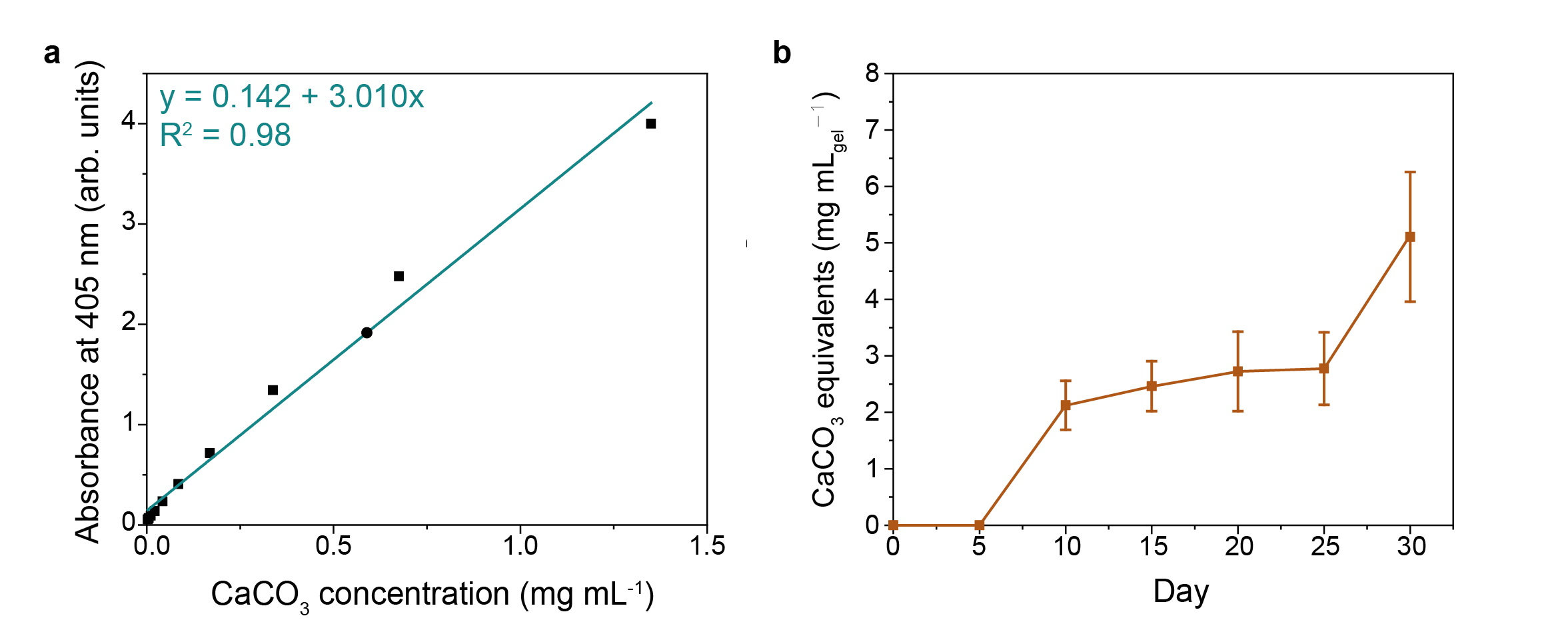


# Supplementary Figure 15. CaCO_3_ precipitate quantification by Alizarin Red S staining a) Calibration curve to calculate equivalent CaCO_3_ amount from the absorbance of Alizarin Red S staining. b) Equivalent CaCO_3_ amounts calculated as difference in Alizarin Red S staining of the precipitates in the biotic and abiotic wells (*n* = 3 randomly selected samples, biological replicates) normalized per milliliter of hydrogel. The printed disc samples were transferred into new well plates every 5 days and Alizarin Red S quantification was done on the recovered well plates. Source data are provided in the Source Data file.


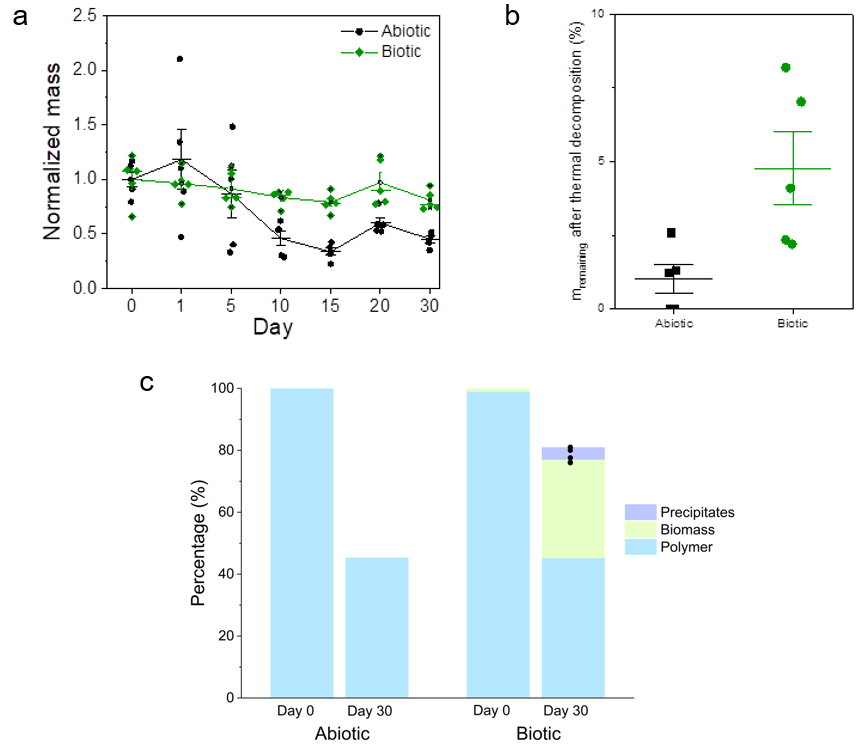


# Supplementary Figure 16. Composition of biotic and abiotic samples. a) Normalized dry mass of abiotic (black, n = 5 randomly selected samples, biological replicates) and biotic (green, *n* = 5 randomly selected samples, biological replicates) samples over the incubation period of 30 days. The dry mass of each sample was normalized to the average initial mass of day 0. The non-cross-linked F127 diffused out over time and caused higher sample to sample variation in dry mass on day 0 to 5. b) Remaining mass of dry abiotic (black) or biotic (green) samples (*n_samples_*=5, biological replicates) after thermal decomposition at 600 °C. c) Composition of abiotic and biotic samples on day 0 and day 30. The dry mass of both abiotic and biotic day 30 samples was normalized to the mass on day 0 (*n* = 5 samples, biological replicates). Biomass percentage was obtained by subtracting the mean precipitates mass percentage from the total dry mass of biomass and precipitates. The decrease in mass in the abiotic samples was due to the diffusion of unfunctionalized Pluronic F127 into the cell culture medium. Source data are provided in the Source Data file.


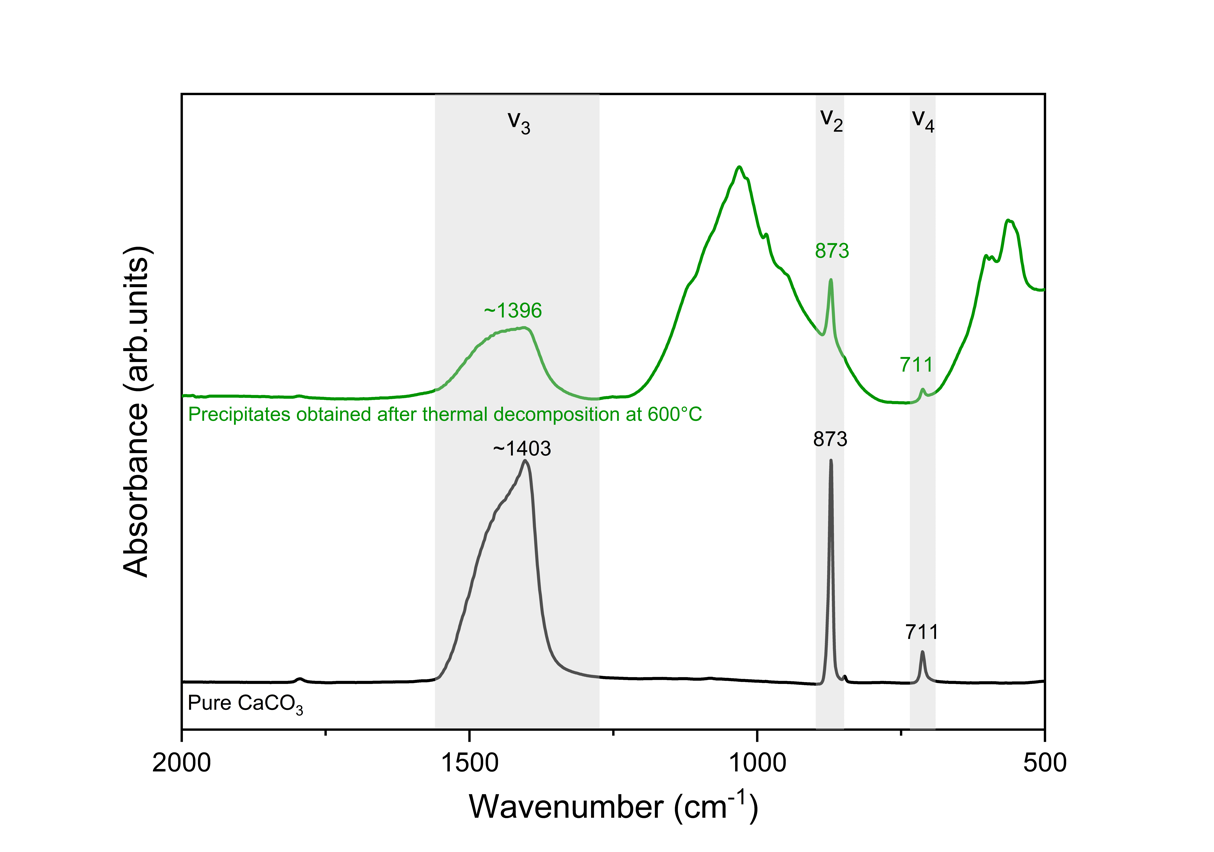


# Supplementary Figure 17. Fourier transform infrared spectroscopy (FTIR) spectra of pure CaCO_3_ (black) and the precipitates after one year of incubation (green) showing indicative peaks of carbonates at 873 and 711 cm^-1^. For inorganic carbonates ν_3_, represents asymmetrical stretching while ν_2_ and ν_4_ indicate in-plane and out-of-plane bending, respectively^[3](https://sciwheel.com/work/citation?ids=15074189&pre=&suf=&sa=0&dbf=0)^. Source data are provided in the Source Data file.


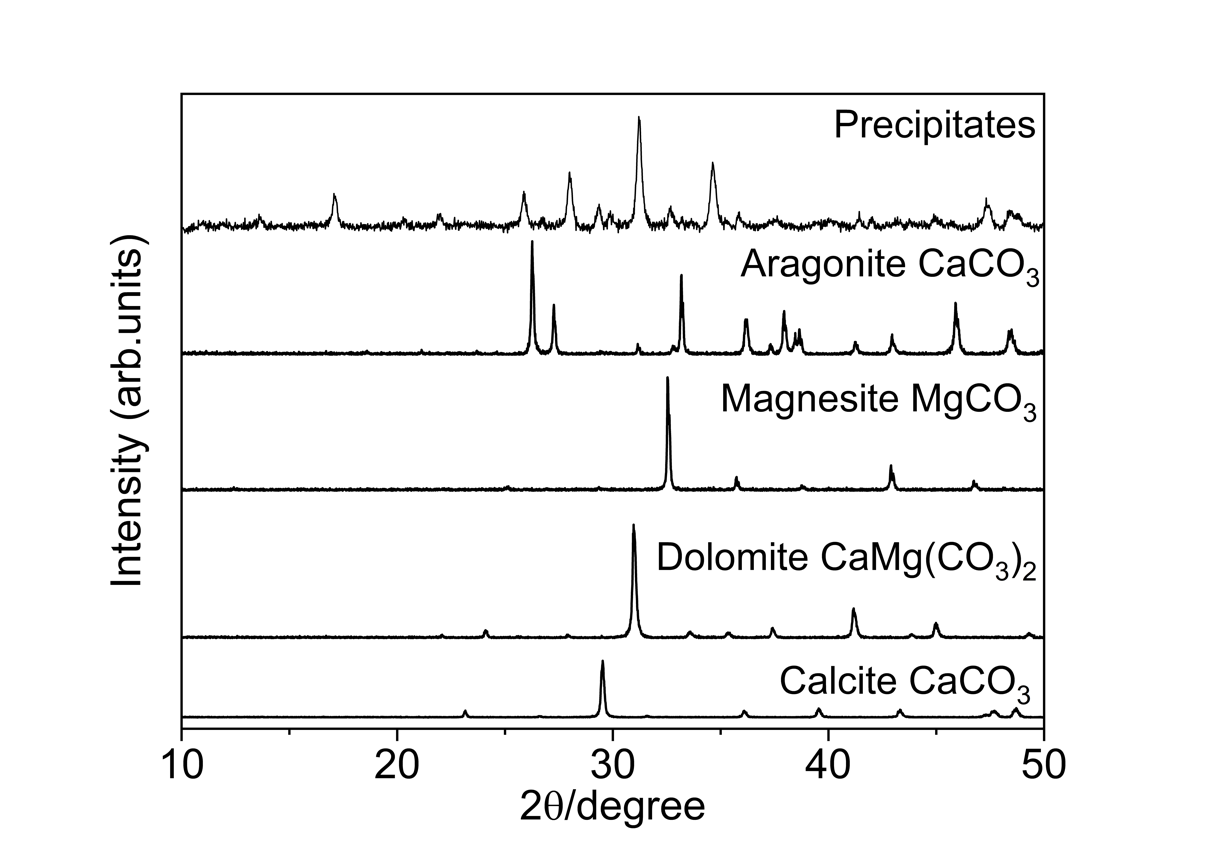


# Supplementary Figure 18. X-ray diffraction (XRD) pattern of the precipitates after sample thermal decomposition at 600 °C and reference XRD patterns of aragonite, magnesite, dolomite and calcite^[7](https://sciwheel.com/work/citation?ids=17001613&pre=&suf=&sa=0&dbf=0)^. Source data are provided in the Source Data file.


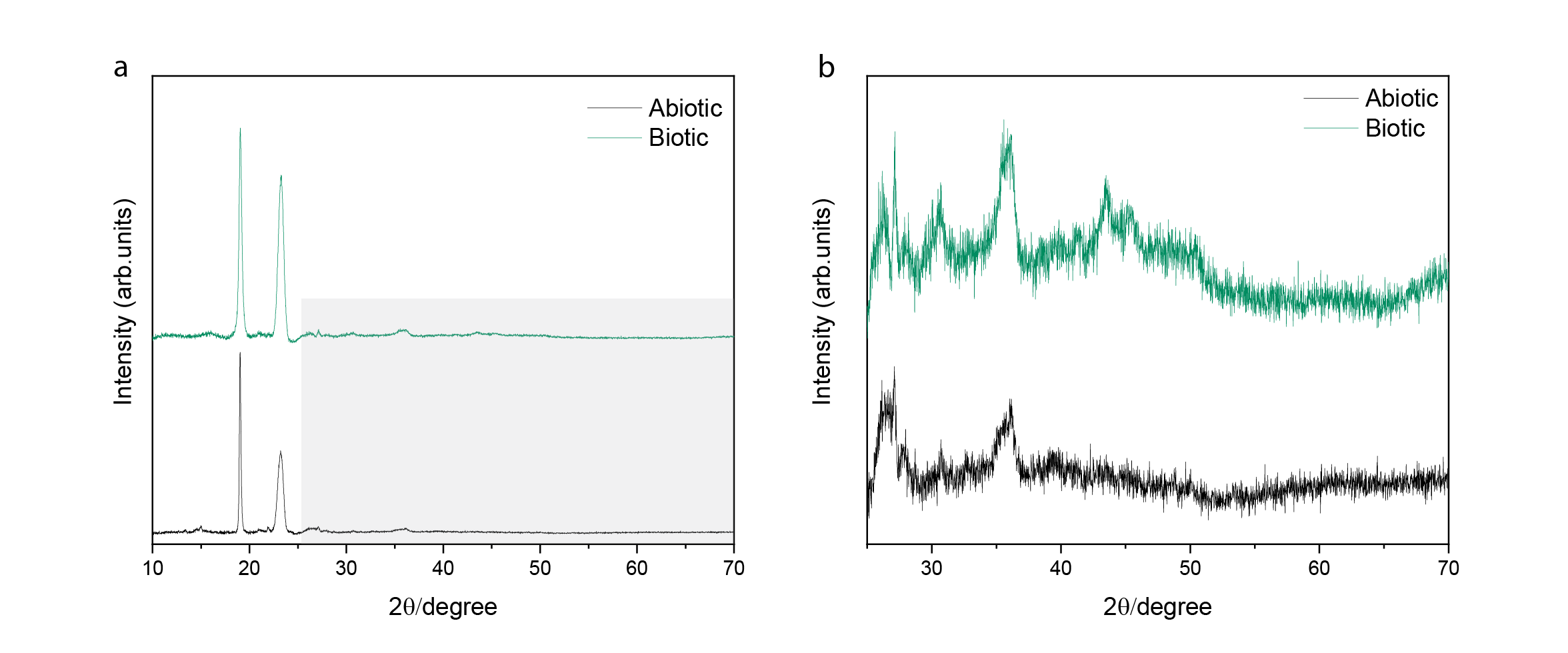


# Supplementary Figure 19. X-ray diffraction (XRD) analysis of the abiotic and biotic samples after 30 days of incubation. The samples were first lyophilized to remove any water content, followed by homogenizing in 1 mL of absolute ethanol for 2 minutes. The major peaks at 18 and 23 correspond to the Pluronic F127 micelles and the calcite peaks are not prominent due to the strong signal from the F127 micelles. a) XRD pattern over 2*θ* of 10 - 70°, b) zoomed-in XRD pattern between a 2θ of 25 and 70°. The signal from the F127-based matrix was much stronger than the one of the precipitates and the signal at 31° could be associated with the calcite peak post thermal decomposition. Source data are provided in the Source Data file.


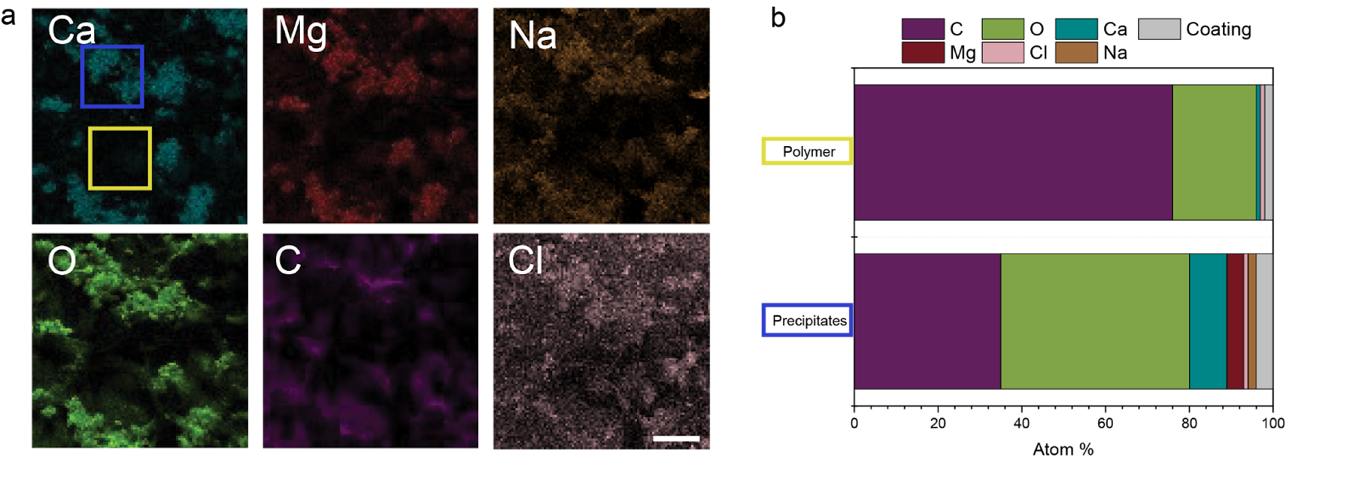


# Supplementary Figure 20. Elemental distribution and atom percentage analysis in biotic samples with precipitates. a) Energy-dispersive X-ray spectroscopy (EDS) elemental mapping of Ca, Mg, Na, O, C, and C atoms in the region with the precipitates of the biotic samples. Representative scanning electron microscope (SEM) image from *n* = 3 independent samples with similar results. Scale bar, 20 μm. b) Atom percentage distribution in polymer- and precipitate- dominant areas of the sample. Source data are provided in the Source Data file.


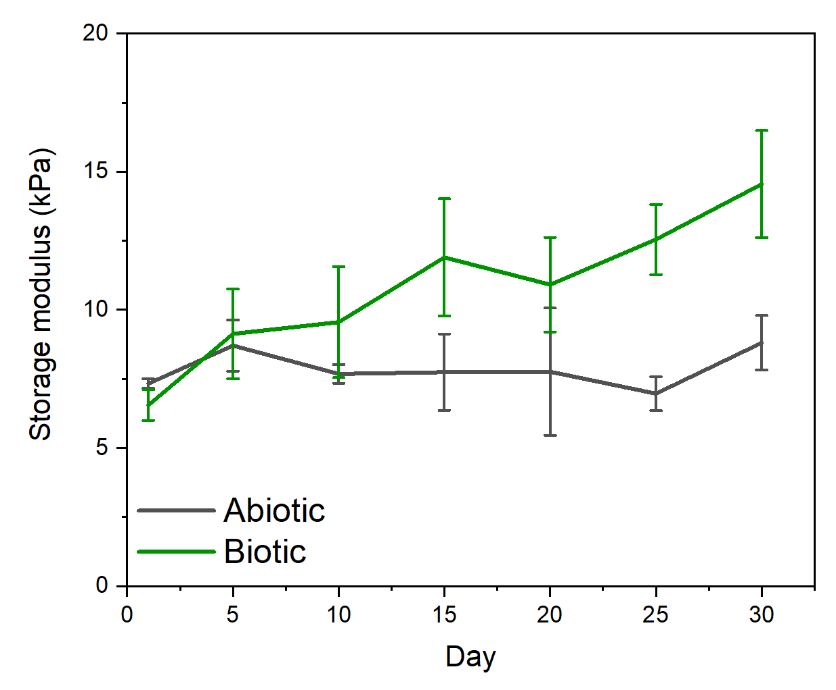


# Supplementary Figure 21. The storage modulus (G′), measured by shear rheometry, of biotic (green) and abiotic (gray) samples during the 30-day incubation period. A significant change in mechanical properties compared with day 1 was also observed after 10 days (*n* = 3 randomly selected samples, biological replicates, *p* = 0.05, p values were calculated using a two-sided two-sample t test, no adjustments were made for multiple comparisons). Source data are provided in the Source Data file.


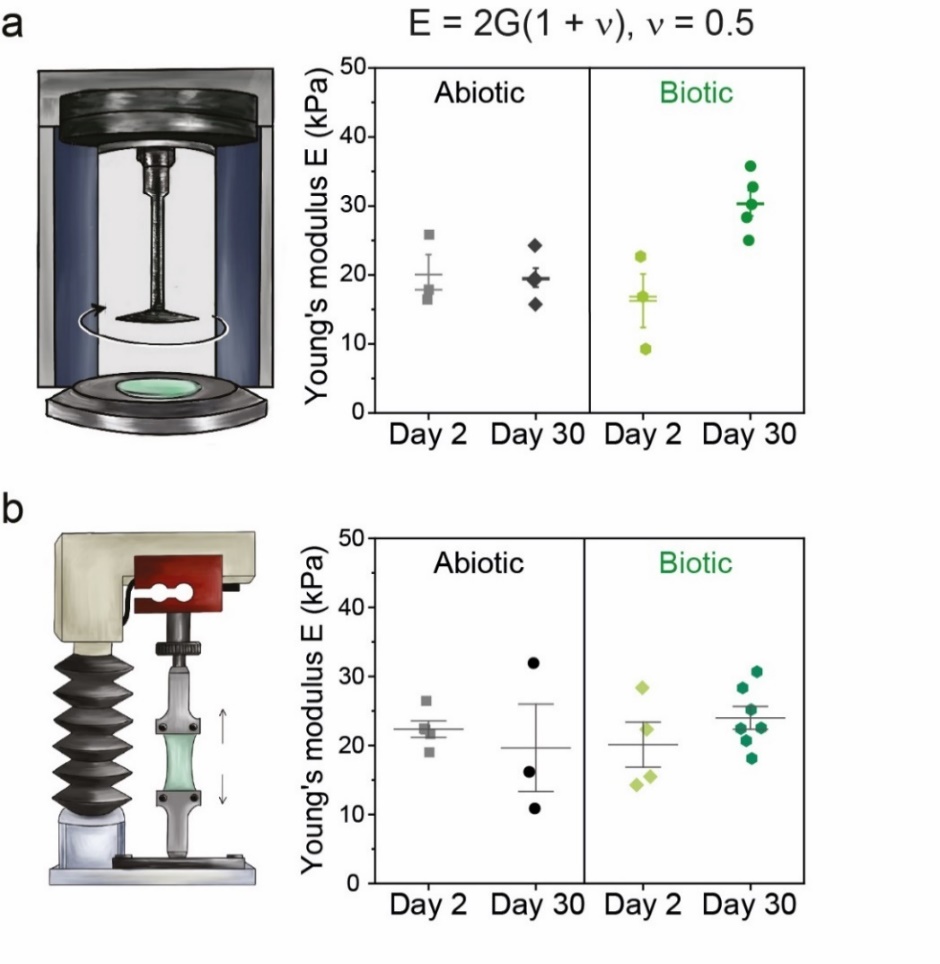


# Supplementary Figure 22. Evaluation of hydrogel mechanical properties. Young’s modulus was a) calculated based on shear modulus obtained with shear rheometer by assuming a Poisson’s ratio of *ν* = 0.5 (*n* = 3 for biotic samples on day 2 & day 30, *n* = 3 for biotic samples on day 2 and *n* = 5 for biotic samples on day 30, biological replicates) and b) obtained by tensile test. Both the tensile test and rheology showed similar Young’s modulus values for the biotic and abiotic samples on day 2 and day 30 (*n* = 4 for abiotic and biotic samples on day 2, *n* = 3 for abiotic samples on day 30 and *n* = 7 for biotic samples on day 30, biological replicates). Cartoon elements used in the schematic diagram were created using Procreate and Adobe Illustrator 2023. Source data are provided in the Source Data file.


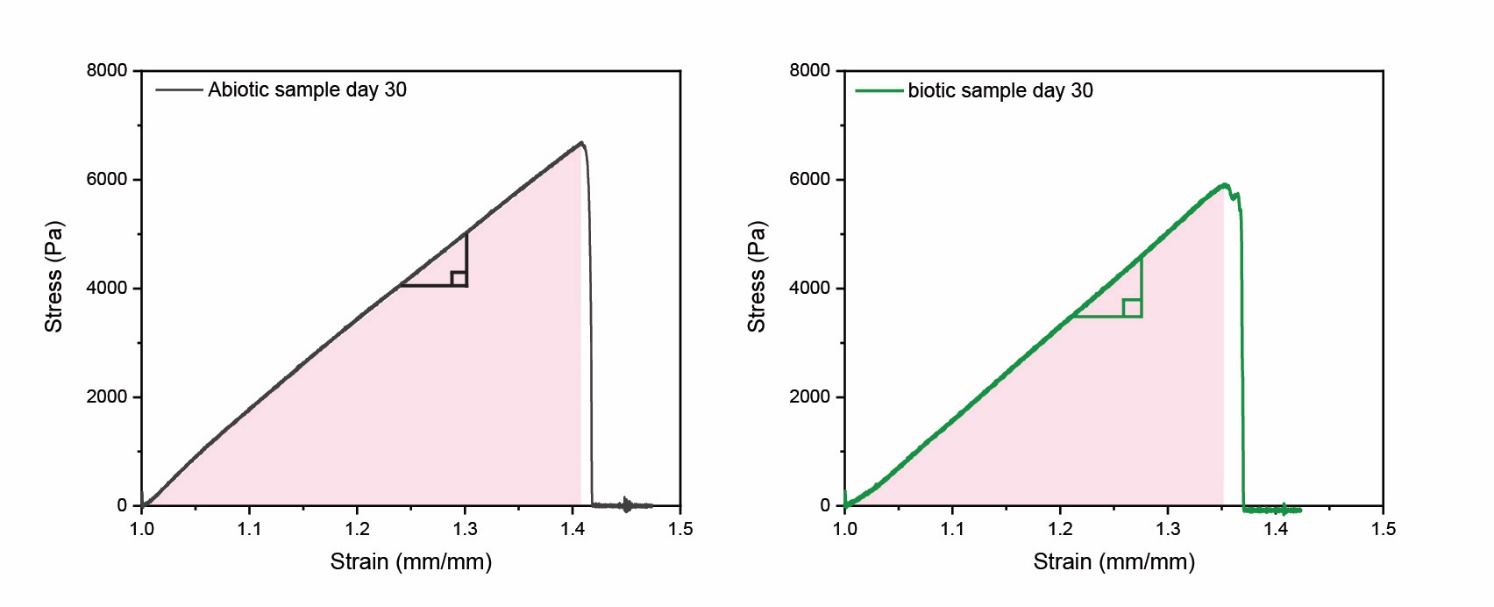


# Supplementary Figure 23. Stress-strain curve of day 30 abiotic (gray) and biotic (green) samples obtained from uniaxial tensile test. The slope of the stress-strain curve was calculated as the Young’s modulus of the samples and the area under the curve shaded in pink was integrated as the material’s toughness. Source data are provided in the Source Data file.


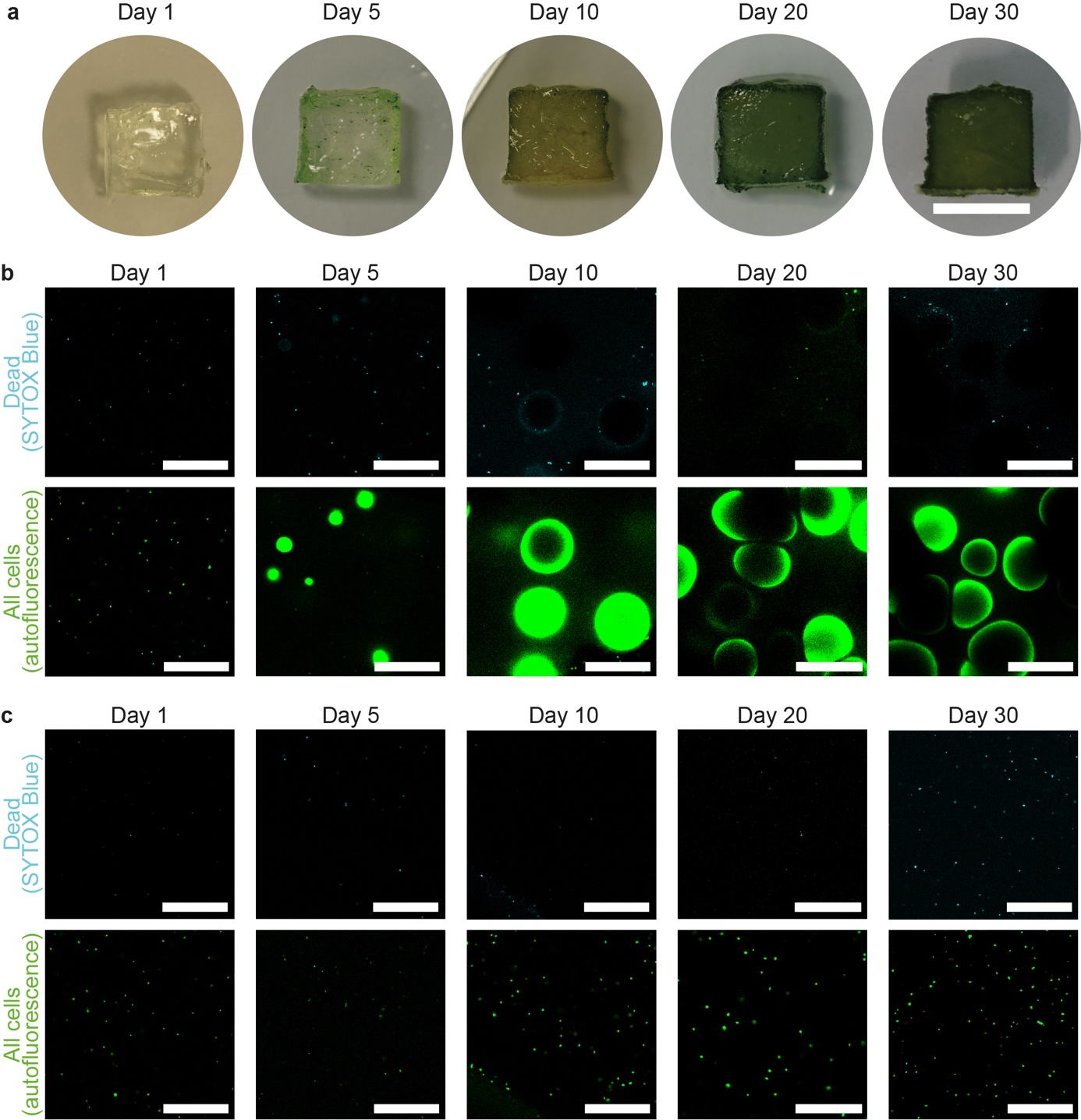


# Supplementary Figure 24. Cell viability analysis of a printed 5 x 5 x 5 mm cube. a) Optical images of the cross section of a 5 x 5 x 5 mm printed cube. Scale bar, 5 mm. Cell viability of samples from b) edge and c) center of a cross section of a 5 x 5 x 5 mm printed cube (SYTOX Blue dead cell staining, cyan; cell autofluorescence, green). Scale bar, 100 μm.


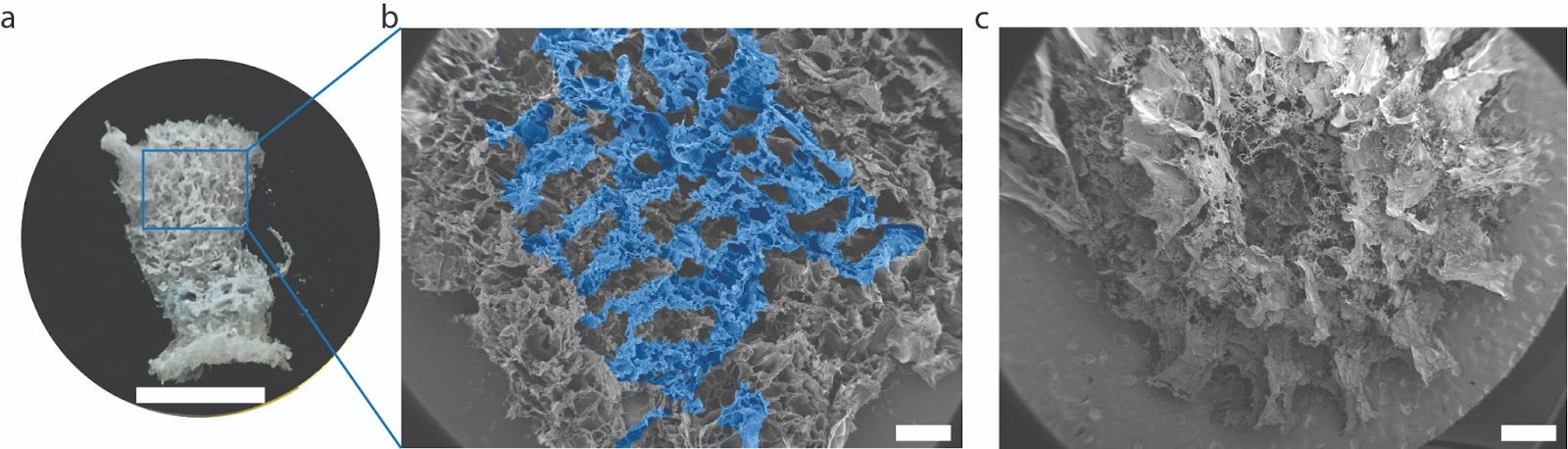


# Supplementary Figure 25. Porosity of lyophilized structures prepared by volumetric 3D printing. a) Optical image of a lyophilized sample incubated for 5 days. Scale bar, 1 cm. b) scanning electron microscope (SEM) image of the sample surface with lattice structure false colored in blue. Scale bar, 1 mm. c) SEM image of the transverse cross-section of the sample prepared by volumetric 3D printing. Scale bar, 1 mm.


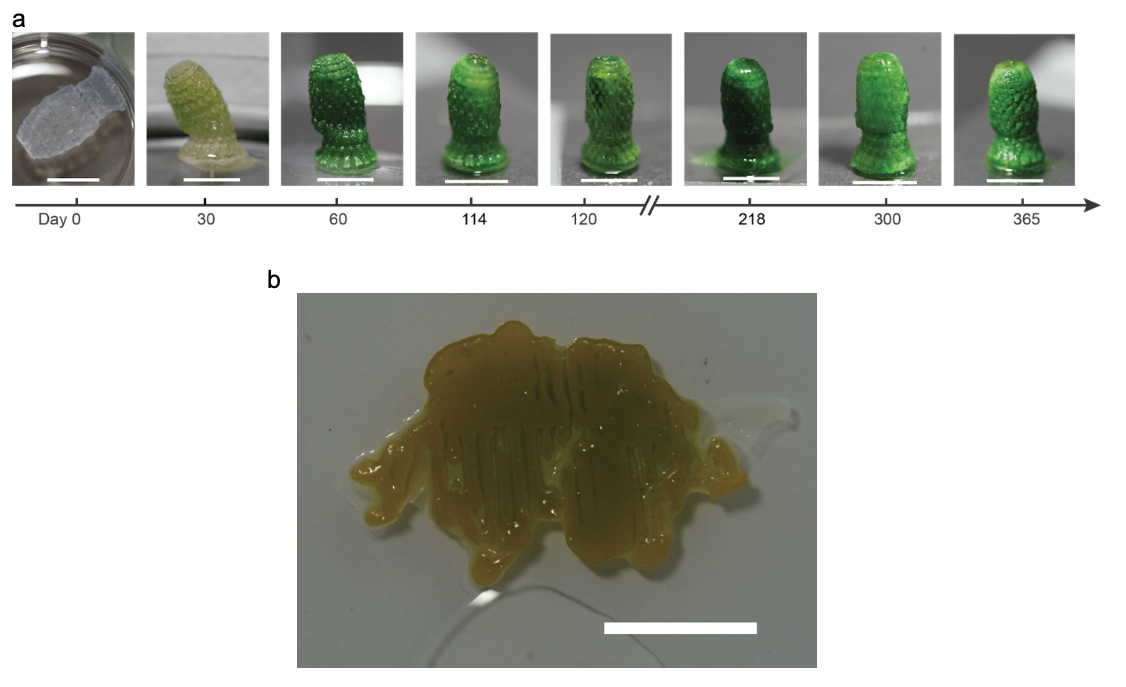


# Supplementary Figure 26. Living material viability under different conditions. a) Optical images of 3D printed photosynthetic living material at different time points up to 365 days. Scale bar, 1 cm. b) 3D-printed photosynthetic living material yellowed within 48 h upon nutrient depletion, indicative of cyanobacteria death and chlorophyll a oxidation. Scale bar, 1 cm.

a


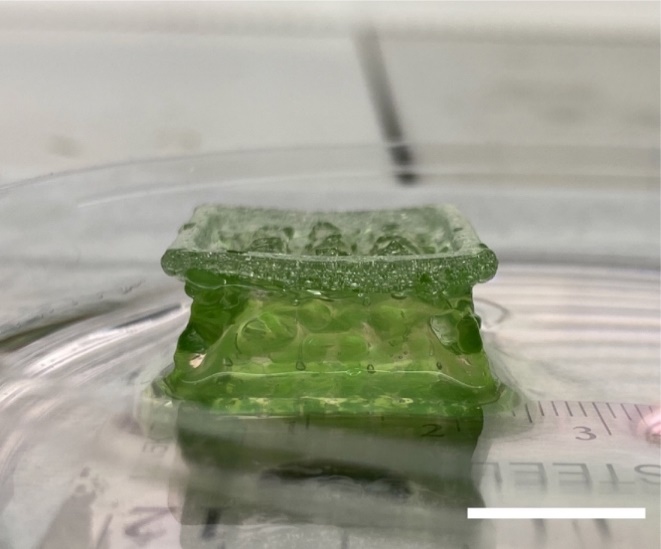


b


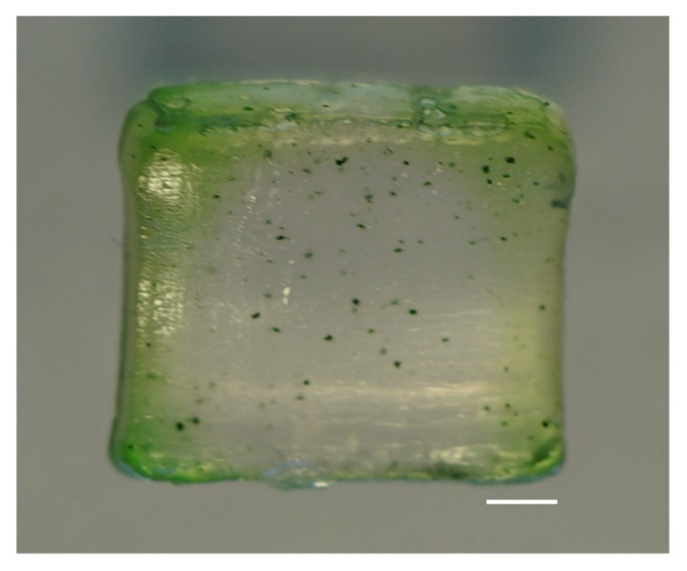


# Supplementary Figure 27. Properties of printed living structures. a) Designed body-centred cubic (BCC) structure that actively transports medium upwards. Scale bar, 1 cm. b) The maximum thickness of the living material to maintain bacteria viability was found to be approximately 2.5 mm from each side (indicated by the green color) and thus a total depth of 5 mm from both sides. Scale bar, 2 mm.

| **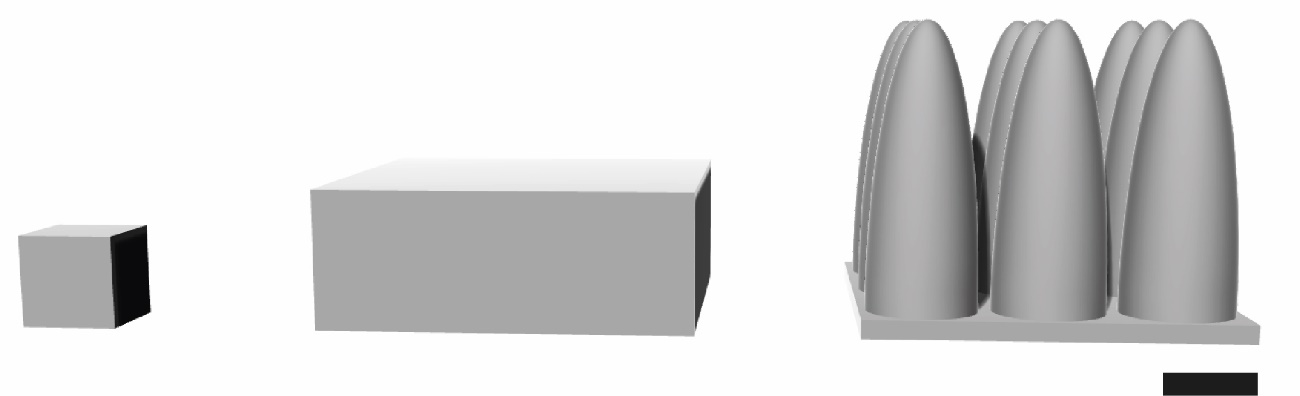** | | |
| --- | --- | --- |
| 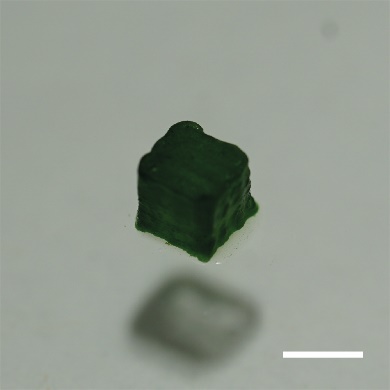 | 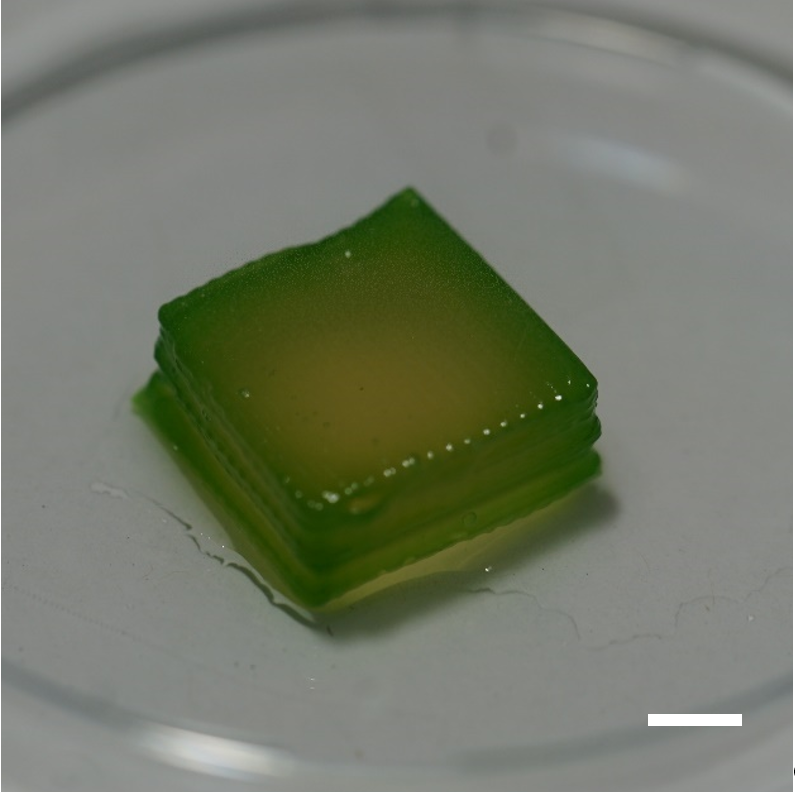 | 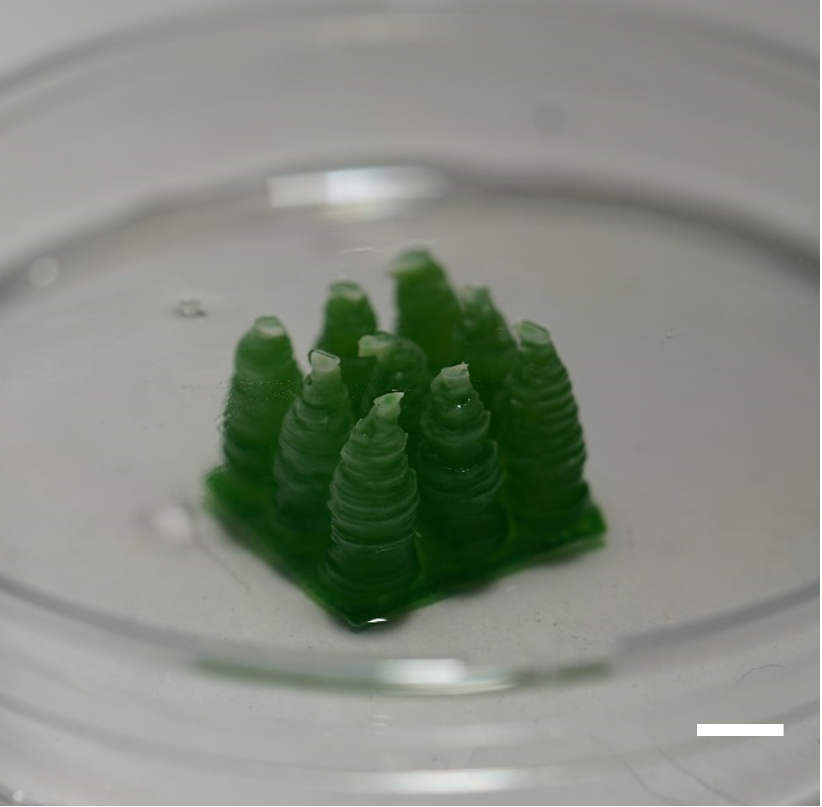 |
| 5 x 5 x 5 mm  0.5 g_material_ cm^-2^  Expressed chlorophyll in ~100% of the volume | 20 x 20 x 7.5 mm block  0.75 g_material_ cm^-2^  Expressed chlorophyll in <<100% of the volume | 20 x 20 mm Pillar array  0.75 g_material_ cm^-2^  Expressed chlorophyll in ~100% of the volume |

# Supplementary Figure 28. 3D models of bulk (left and middle) and pillar array structure (right) and corresponding printed structures. Printed 5 x 5 x 5 mm bulk volume expressed chlorophyll in ~100 % of the volume on day 16. However, the 20 x 20 x 7.5 block did not show evidence of full chlorophyll expression in the structure on day 5 and, therefore, pillar array design was employed to increase the volume of material per area. The pillar array designed with the same material volume and surface coverage expressed chlorophyll in ~100 % of the volume on day 5. Scale bars, 0.5 cm.


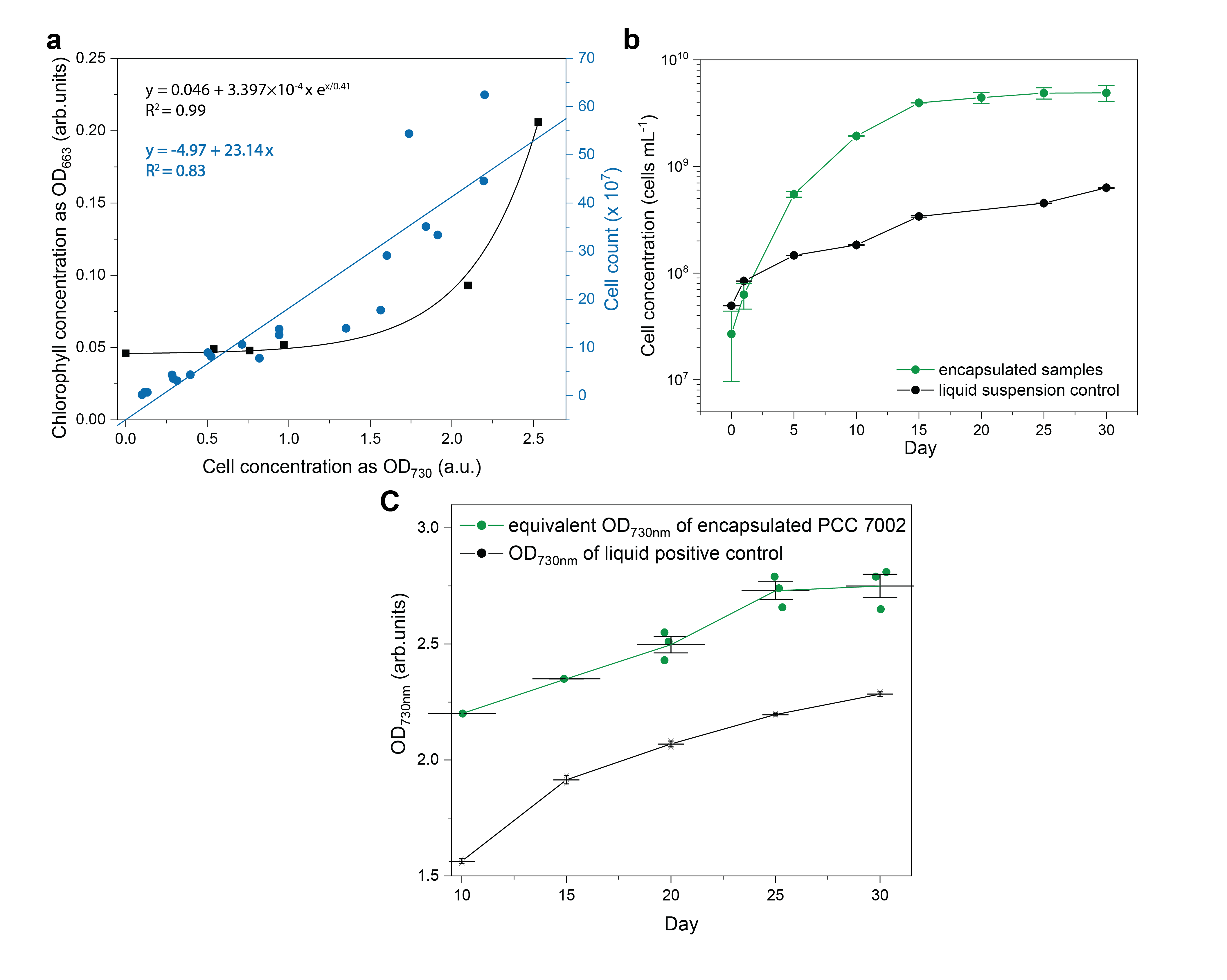


# Supplementary Figure 29. Cell concentration changes during incubation in liquid suspension and encapsulated samples. a) Calibration curve fitting to relate chlorophyll absorption at optical density OD_663nm_ to cell suspension OD_730nm_ (black) and calibration curve fitting to relate cell suspension OD_730nm_ and cell number measured via particle counting (black). b) Cell concentration in liquid suspension (particle counting, black, *n* = 3 samples, biological replicates) compared to average cell concentration in encapsulated samples obtained via confocal microscopy max projection (light green, *n* = 3 confocal max projection images from randomly selected samples, biological replicates) and chlorophyll extraction (dark green, *n* = 3 from randomly selected samples, biological replicates). c) Comparison of equivalent OD_730nm_ of encapsulated cells (green, *n* = 3, biological replicates) and actual OD_730nm_ of liquid suspension (black, *n* = 3, biological replicates) from day 10. Source data are provided in the Source Data file.


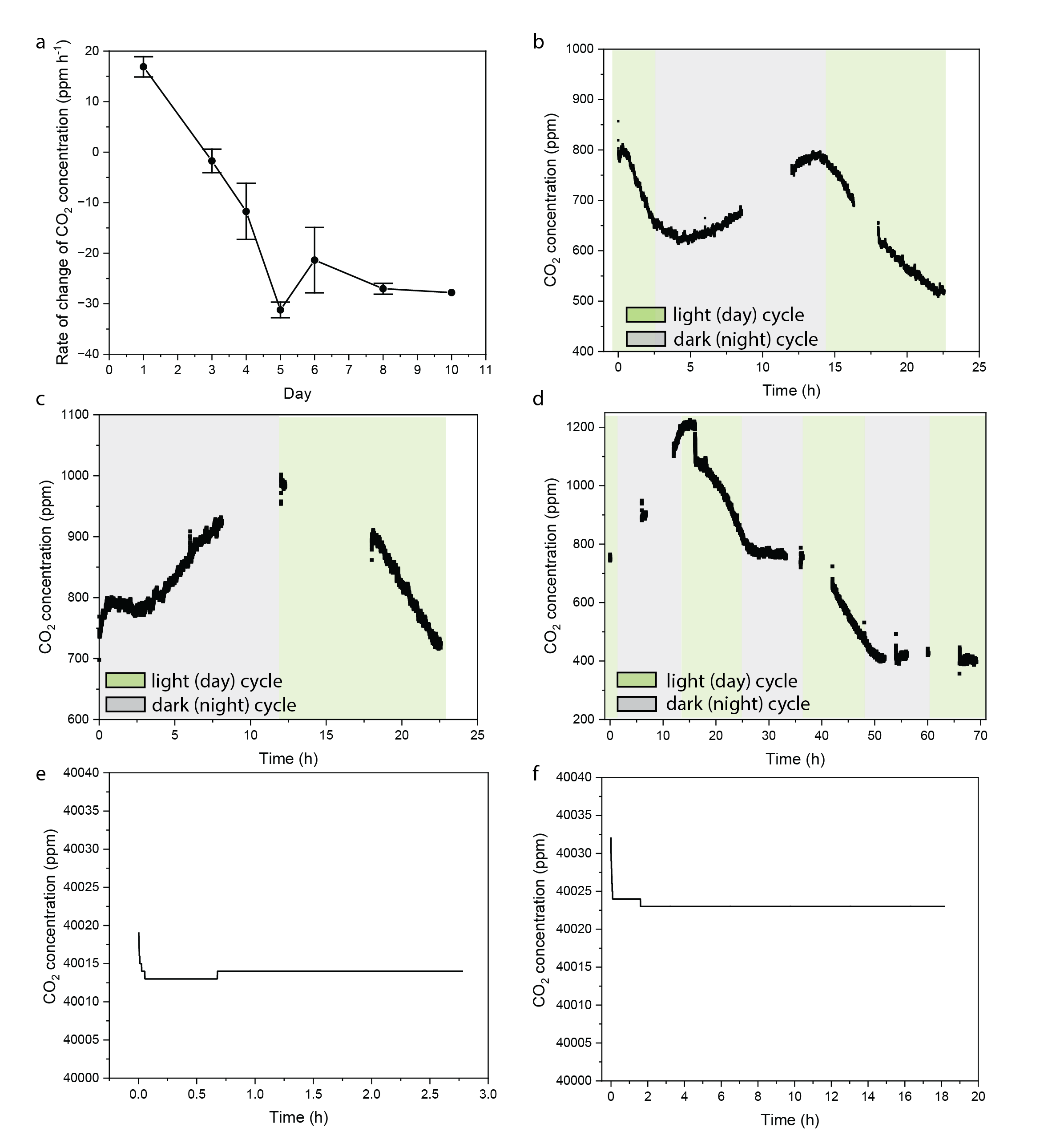


# Supplementary Figure 30. CO_2_ concentration changes and sequestration in a closed container over time. a) Rate of change of CO_2_ concentration in a 250 mL closed container over a measurement period of 10 days with a 0.25 mL sample and 10 mL culture medium (*n* = 2 rounds of measurements, biological replicates). CO_2_ sequestration was observed from day 3 and plateaued beyond day 5. b),c),d) Representative snapshots of CO_2_ evolution in a closed container over 1 day-night cycle and 3 day-night cycles. Black scatters: CO_2_ concentration; green shading: light (day) conditions; grey shading: dark (night) conditions. e), f) CO_2_ leakage tests in 250 mL closed containers without cells loaded with an excess concentration of CO_2_*.* Source data are provided in the Source Data file.


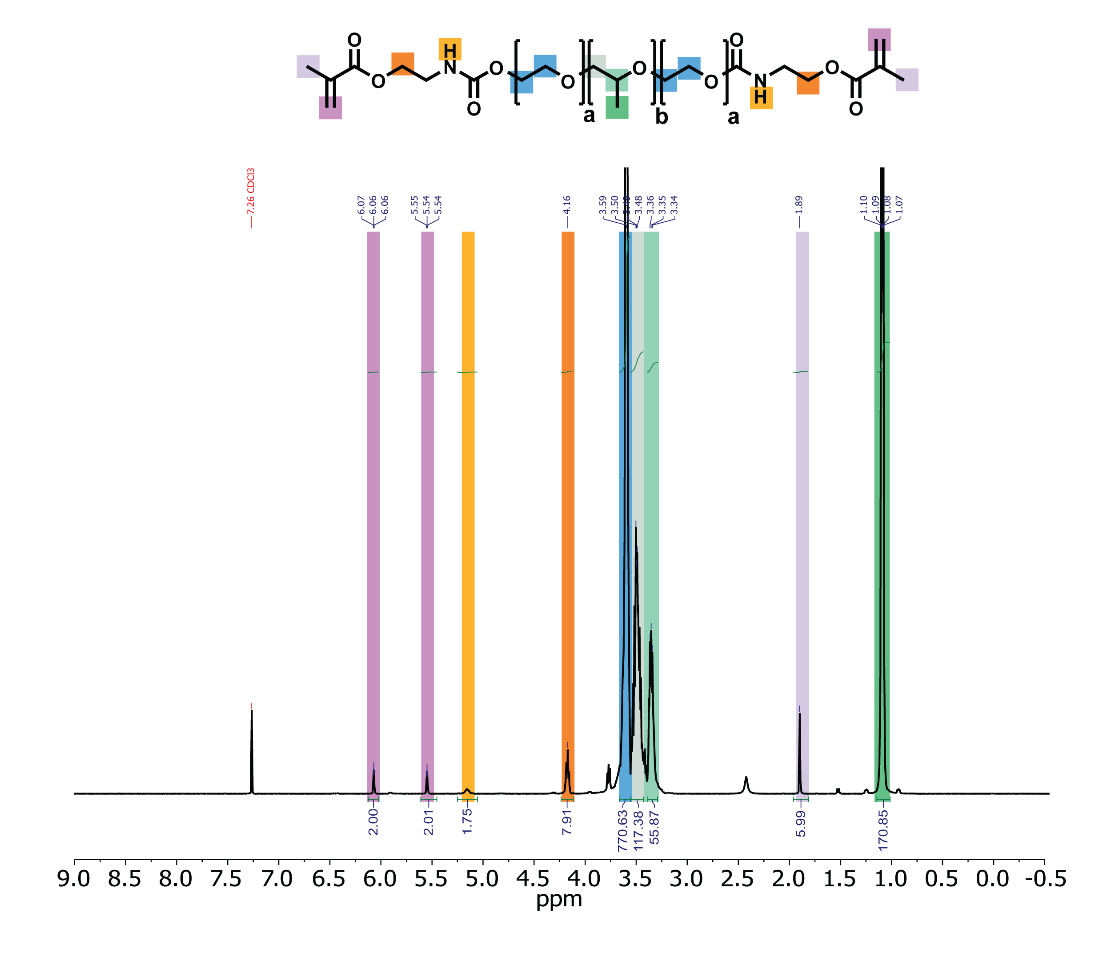


# Supplementary Figure 31. ^1^H-NMR spectrum of F127-BUM in CDCl_3_. Proton assignment was visualized by highlighting with the same color the specific protons in the chemical structure and their associated signals in the spectra.


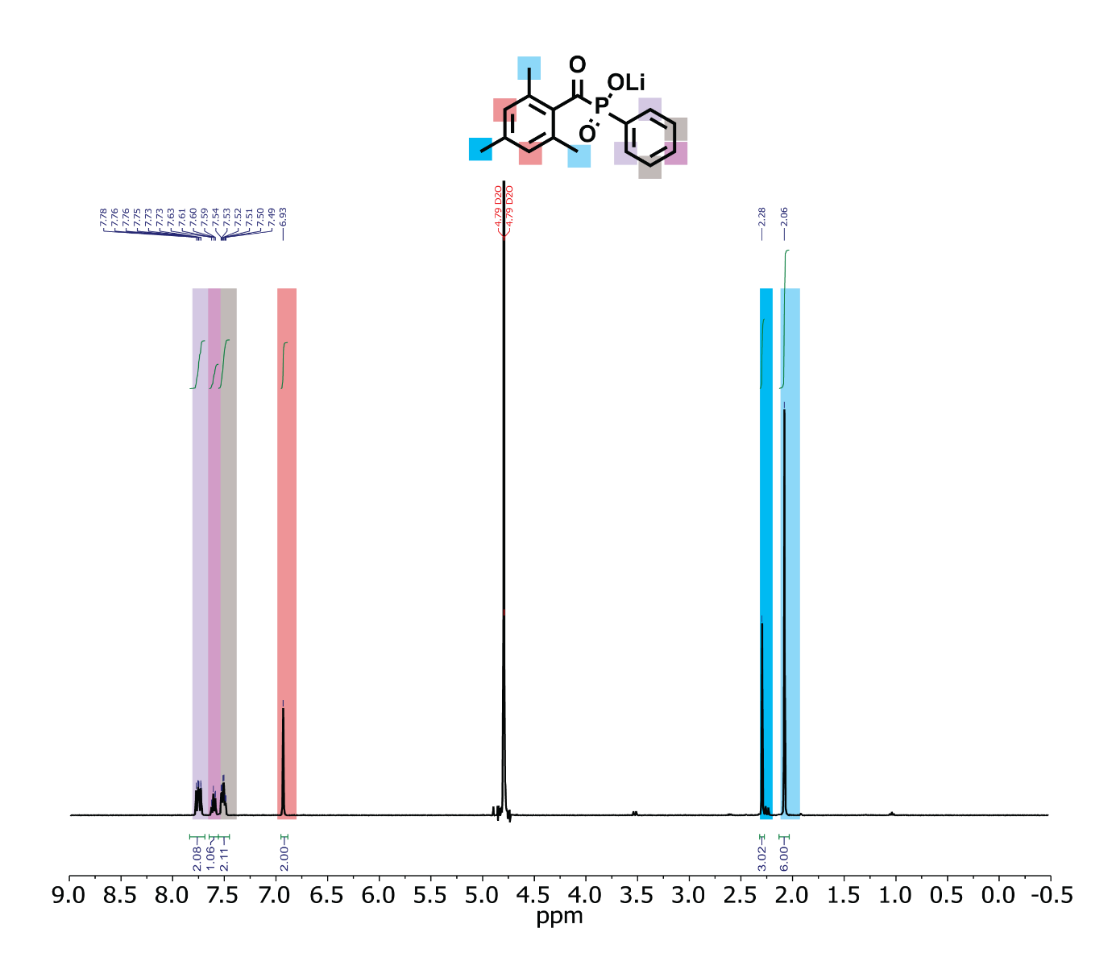


# Supplementary Figure 32. 1H-NMR spectrum of LAP in D_2_O. Proton assignment was visualized by highlighting with the same color the specific protons in the chemical structure and their associated signals in the spectra.


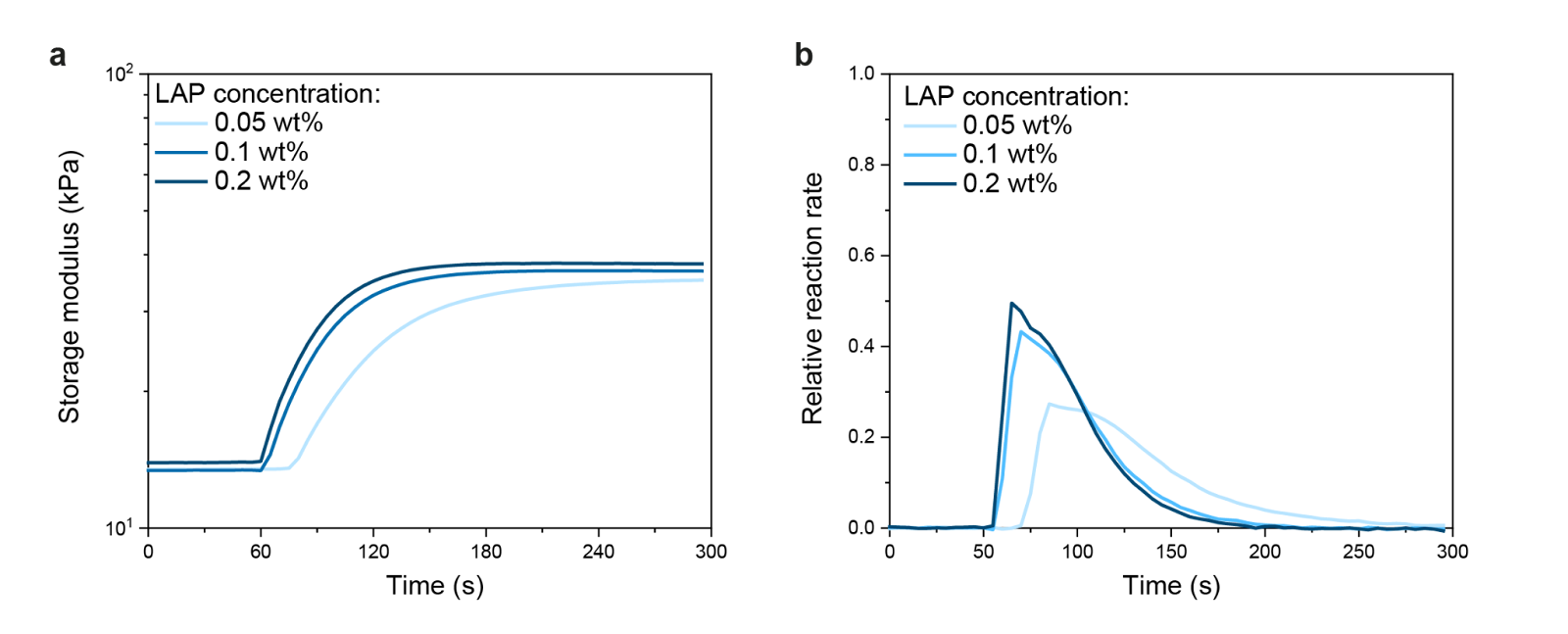


# Supplementary Figure 33. Photoinitiator concentration screening. a) Photo-cross-linking of the bioink with different photoinitiator lithium phenyl-2,4,6-trimethylbenzoylphosphinate (LAP) concentrations (wavelength *λ* = 405 nm; intensity *I* = 8 mW cm^-2^; light turned on at time *t* = 60 s). b) relative reaction rates of the same hydrogels, calculated based on the time derivative of the storage modulus. Source data are provided in the Source Data file.


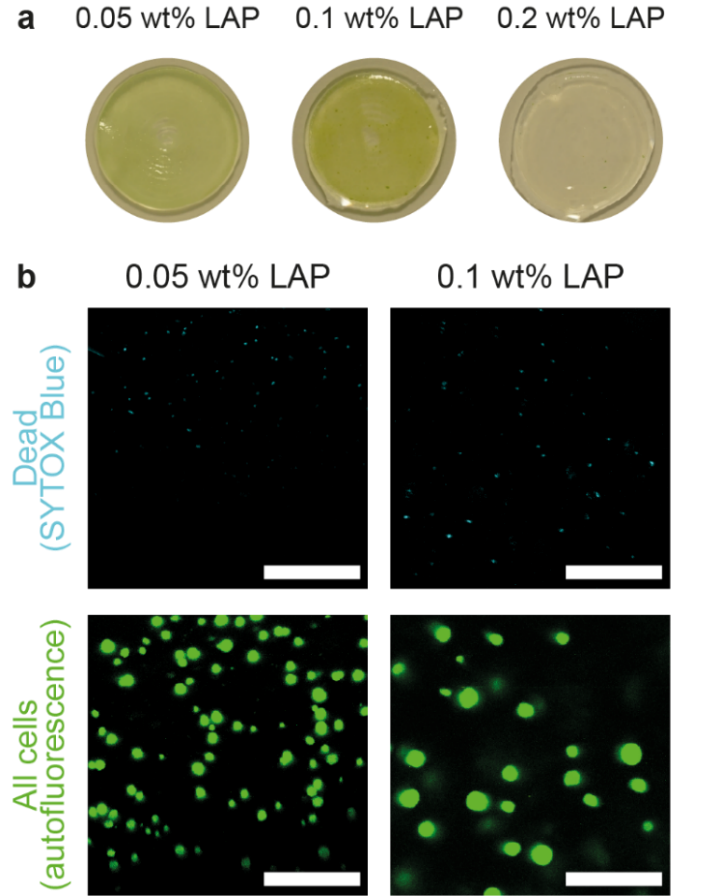


# Supplementary Figure 34. Cell viability with different photoinitiator concentrations. a) Differences in cell growth represented as green color of the living samples on day 5 after photo-cross-linking the bioink at with 0.05, 0.1, and 0.2 wt% lithium phenyl-2,4,6-trimethylbenzoylphosphinate (LAP). The lowest cell growth was observed with 0.2 wt% LAP in the latter case. b) Cell viability on day 5 after photo-cross-linking the bioink at with 0.05 and 0.1 wt% LAP (SYTOX Blue dead cell staining, cyan; cell autofluorescence, green; maximum intensity Z-projection). Scale bar, 100 μm.

**
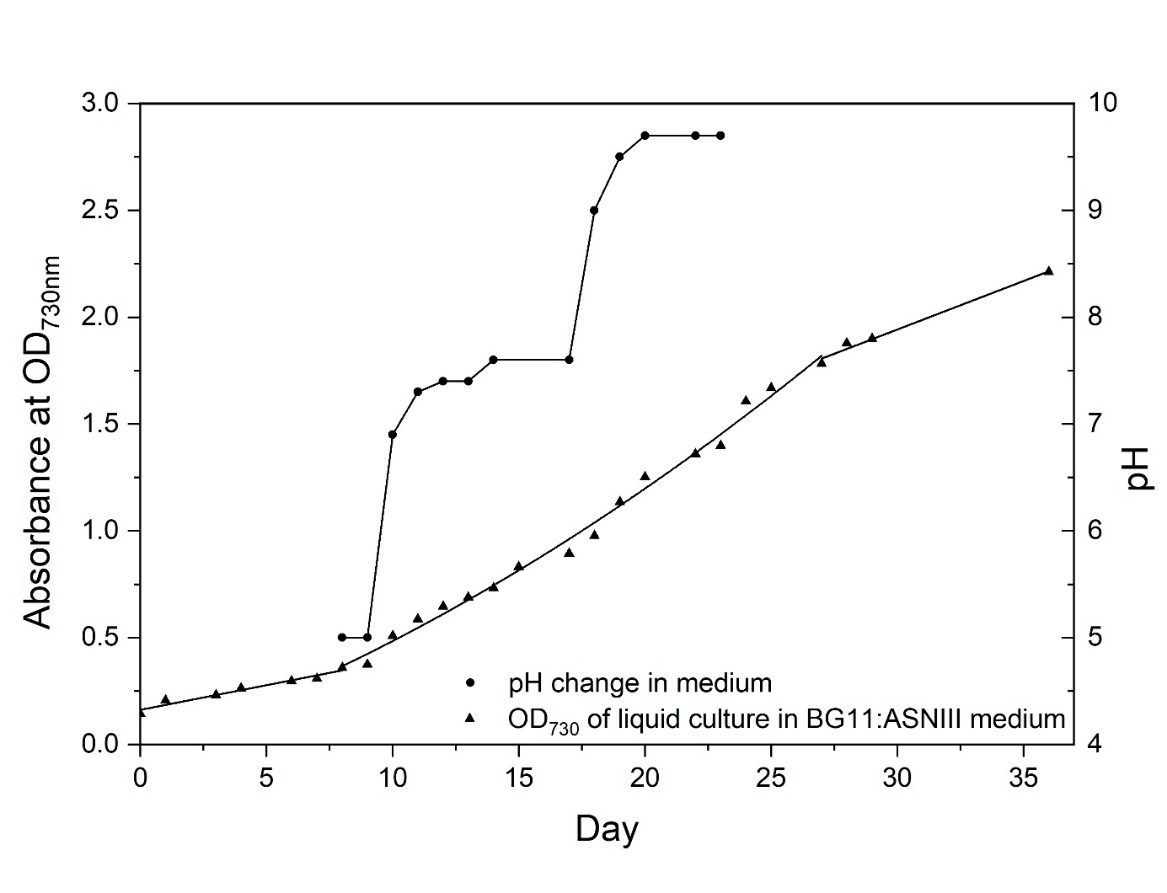
**

# Supplementary Figure 35. Representative growth curve (*n* = 2) of PCC 7002 in BG11–ASNIII medium and pH change of the liquid culture. Source data are provided in the Source Data file.


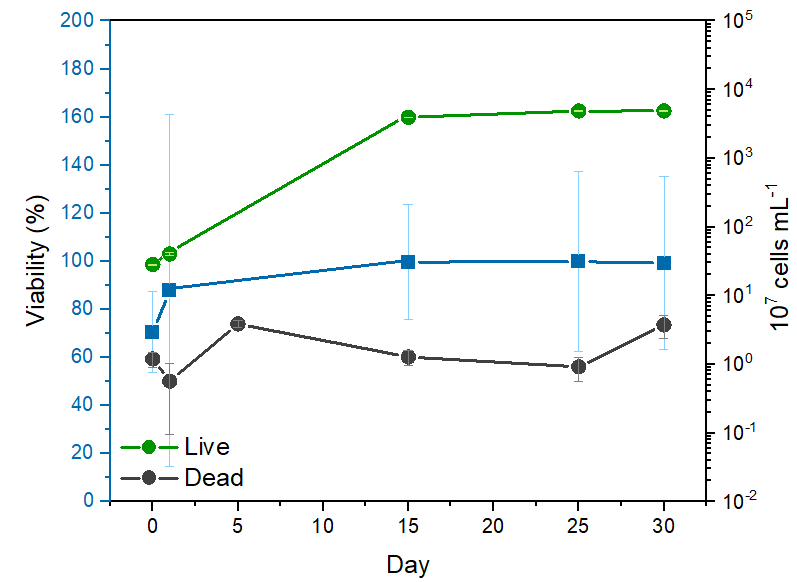


# Supplementary Figure 36. Cell viability (blue) and comparison of live (green, *n* = 3) and dead (black; *n* = 3) cell concentration in encapsulated disc samples obtained via confocal microscopy max projection (day 0, day 1, and all dead cell data) or chlorophyll extraction (live cells day 15–30). Source data are provided in the Source Data file.

# Supplementary Tables

# Supplementary Table 1. Hydrogel shear-thinning parameters, Source data are provided in the Source Data file.

| Sample | *K*, Pa s^-n^ | *n* | r^2^ |
| --- | --- | --- | --- |
| Abiotic hydrogel | 287±7 | 0.091±0.005 | 0.99 |

# Supplementary Table 2. Compressive modulus of photosynthetic living materials after 400 days of incubation

| s/n | Sample thickness [mm] | Compressive modulus [kPa] |
| --- | --- | --- |
| 1 | 3.871 | 120.9 |
| 2 | 3.966 | 105.1 |
| 3 | 3.218 | 102.8 |
| 4 | 3.727 | 112.8 |
| 5 | 3.765 | 113.7 |

# Supplementary Table 3. CO_2_ sequestration per gram of hydrogel material after 400 days of incubation, the mass of CaCO_3_ precipitates were obtained after thermal decomposition at 600 °C

| s/n | Sample wet mass [mg] | CaCO_3_ mass [mg] | CO_2_ sequestration per gram of hydrogel material [mg · g^-1^] |
| --- | --- | --- | --- |
| 1 | 206 | 339.9 | 34.0 |
| 2 | 60.4 | 215.5 | 21.6 |
| 3 | 64.7 | 258.3 | 25.8 |
| 4 | 114.5 | 355.9 | 30.4 |

# Supplementary Table 4. BG11-ASNIII medium components

| Component | Concentration [g L^-1^] |
| --- | --- |
| NaCl | 25.0 |
| MgSO_4_ anhydrous | 1.78036 |
| KCl | 0.50 |
| NaNO_3_ | 2.265 |
| MgCl_2_ · 6H_2_O | 2.00 |
| K_2_HPO_4_ anhydrous | 0.04331 |
| CaCl_2_ anhydrous | 0.4053 |
| Citric Acid | 0.009056 |
| Ferric ammonium citrate | 0.00906 |
| EDTA (disodium magnesium) | 0.00101 |
| Na_2_CO_3_ | 0.0402 |
| MilliQ water | To 1L |
| After autoclaving |  |
| A5+ Co Trace metals | 1 mL |
| Vitamin B12 | 0.010 |

# Supplementary Table 5. A5+ Co Trace metal medium components

| Component | Concentration [g L^-1^] |
| --- | --- |
| H_3_BO_3_ | 2.86 |
| MnCl_2_ · 4H_2_O | 1.81 |
| ZNSO_4_ · 7H_2_O | 0.222 |
| Na2MoO_4_ · 2H_2_O | 0.39 |
| CuSO_4_ · 5H_2_O | 0.079 |
| Co(NO_3_)_2_ · 6H_2_O | 0.0494 |

# Supplementary References

[1.    Rounds, S. A. & Wilde, F. D. *Chapter A6. Section 6.6. Alkalinity and acid neutralizing capacity*. (2012).](https://sciwheel.com/work/bibliography/17001610)

[2.    Williams, W. D. Conductivity and salinity of Australian salt lakes. *Mar. Freshwater Res.* **37**, 177 (1986).](https://sciwheel.com/work/bibliography/17001599)

[3.    Salter, M. A., Perry, C. T. & Smith, A. M. Calcium carbonate production by fish in temperate marine environments. *Limnol. Oceanogr.* **64**, 2755–2770 (2019).](https://sciwheel.com/work/bibliography/15074189)

[4.    Millik, S. C. *et al.* 3D printed coaxial nozzles for the extrusion of hydrogel tubes toward modeling vascular endothelium. *Biofabrication* **11**, 045009 (2019).](https://sciwheel.com/work/bibliography/10883750)

[5.    Majima, T., Schnabel, W. & Weber, W. Phenyl-2,4,6-trimethylbenzoylphosphinates as water-soluble photoinitiators. Generation and reactivity of  O=P(C_6_H_5_)(O-) radical anions. *Wiley* **192**, 2307–2315 (1991).](https://sciwheel.com/work/bibliography/13097264)

[6.    Fairbanks, B. D., Schwartz, M. P., Bowman, C. N. & Anseth, K. S. Photoinitiated polymerization of PEG-diacrylate with lithium phenyl-2,4,6-trimethylbenzoylphosphinate: polymerization rate and cytocompatibility. *Biomaterials* **30**, 6702–6707 (2009).](https://sciwheel.com/work/bibliography/1528628)

[7.    Database of Raman spectroscopy, X-ray diffraction and chemistry of minerals. https://rruff.info/.](https://sciwheel.com/work/bibliography/17001613)
