# Supplementary figures and images for: Dual carbon sequestration with photosynthetic living materials
Source: Nat Commun. 2025 Apr 23;16:3832. doi: 10.1038/s41467-025-58761-y (PMC12019168; doi:10.1038/s41467-025-58761-y)

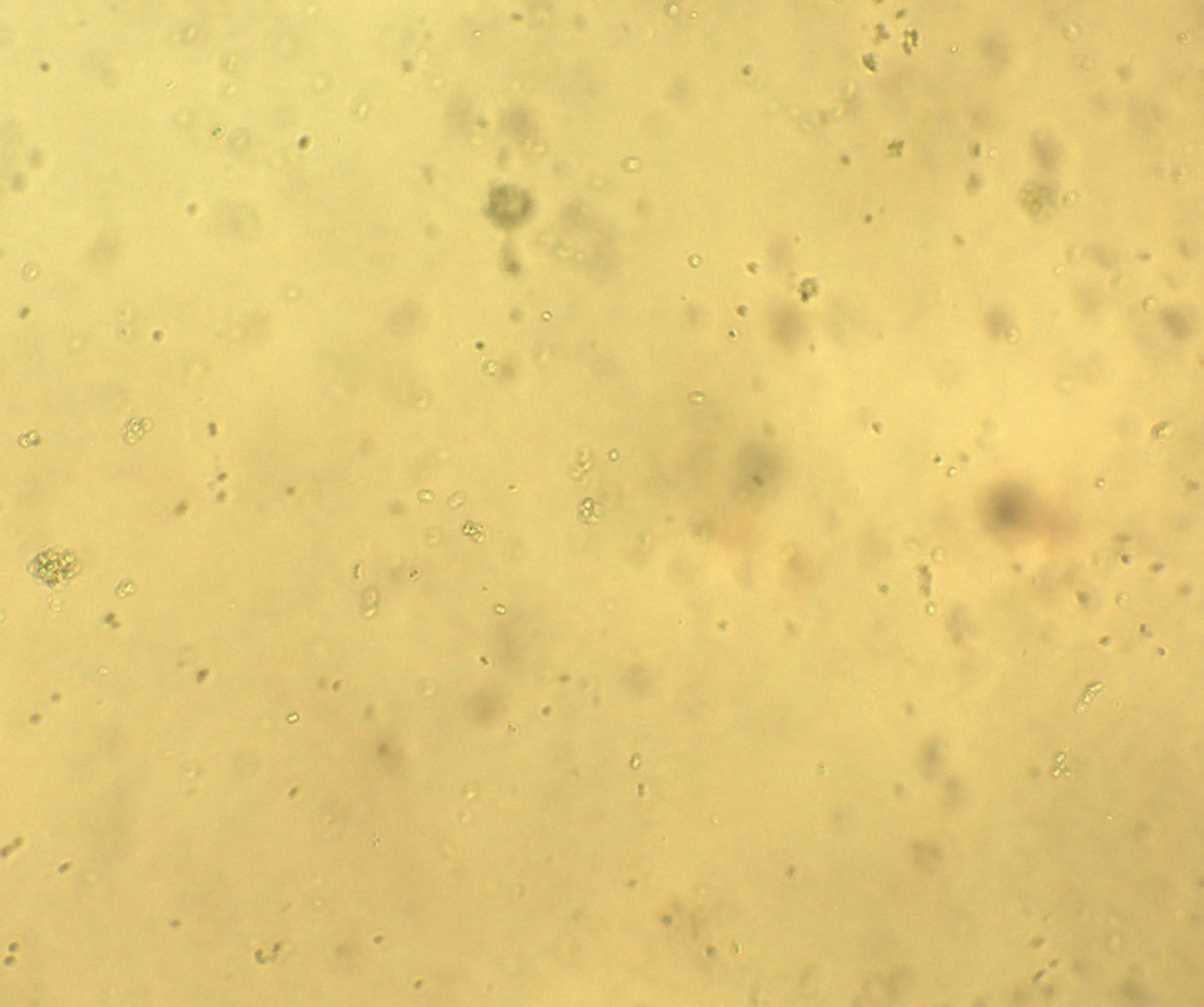

Supplement: Supplementary file 4 — Source Data [file 41467_2025_58761_MOESM4_ESM.zip › Figure 2/biotic day 0 alizarin red 40x.jpeg]

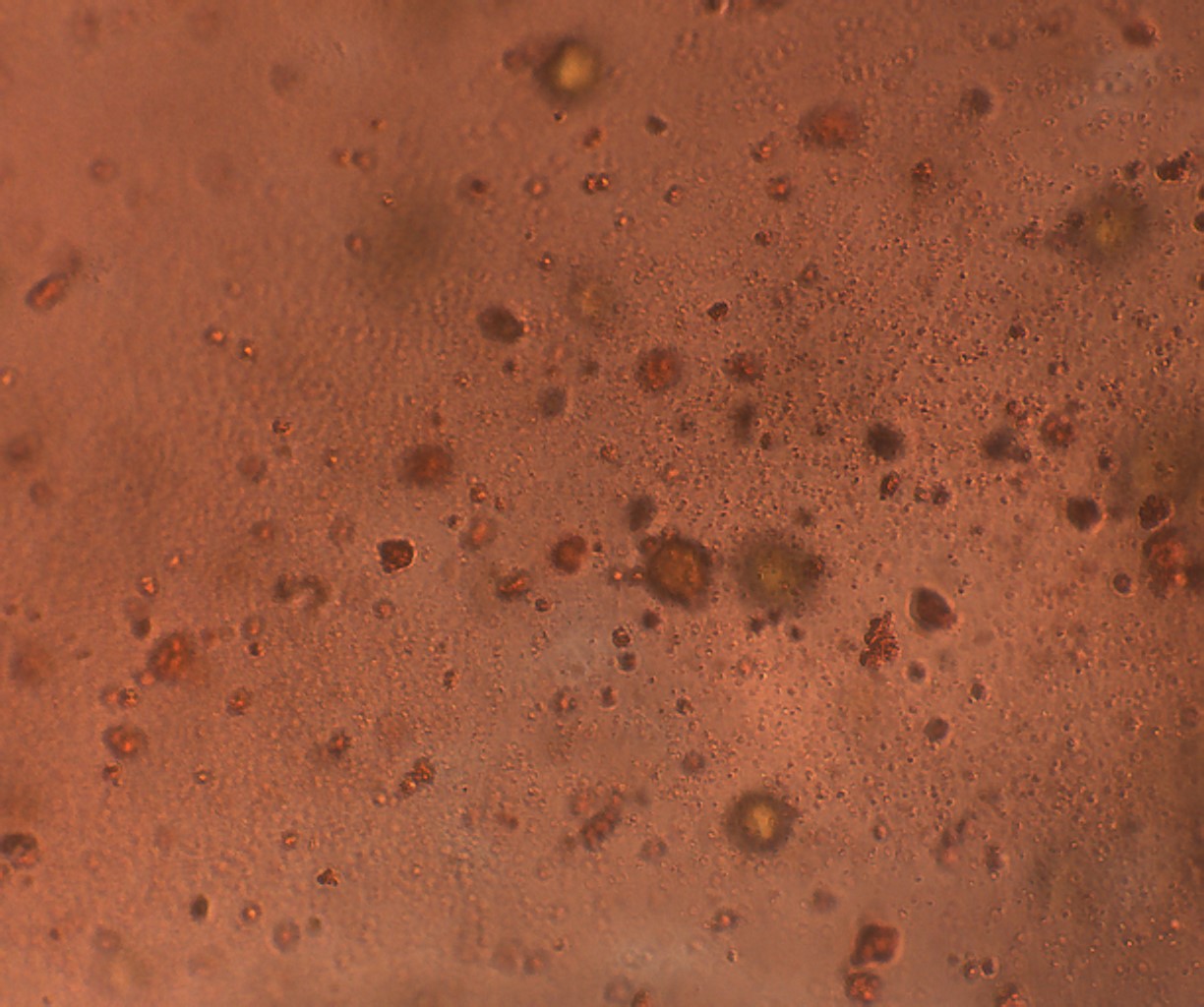

Supplement: Supplementary file 4 — Source Data [file 41467_2025_58761_MOESM4_ESM.zip › Figure 2/biotic day 10 alizarin red 40x.jpeg]

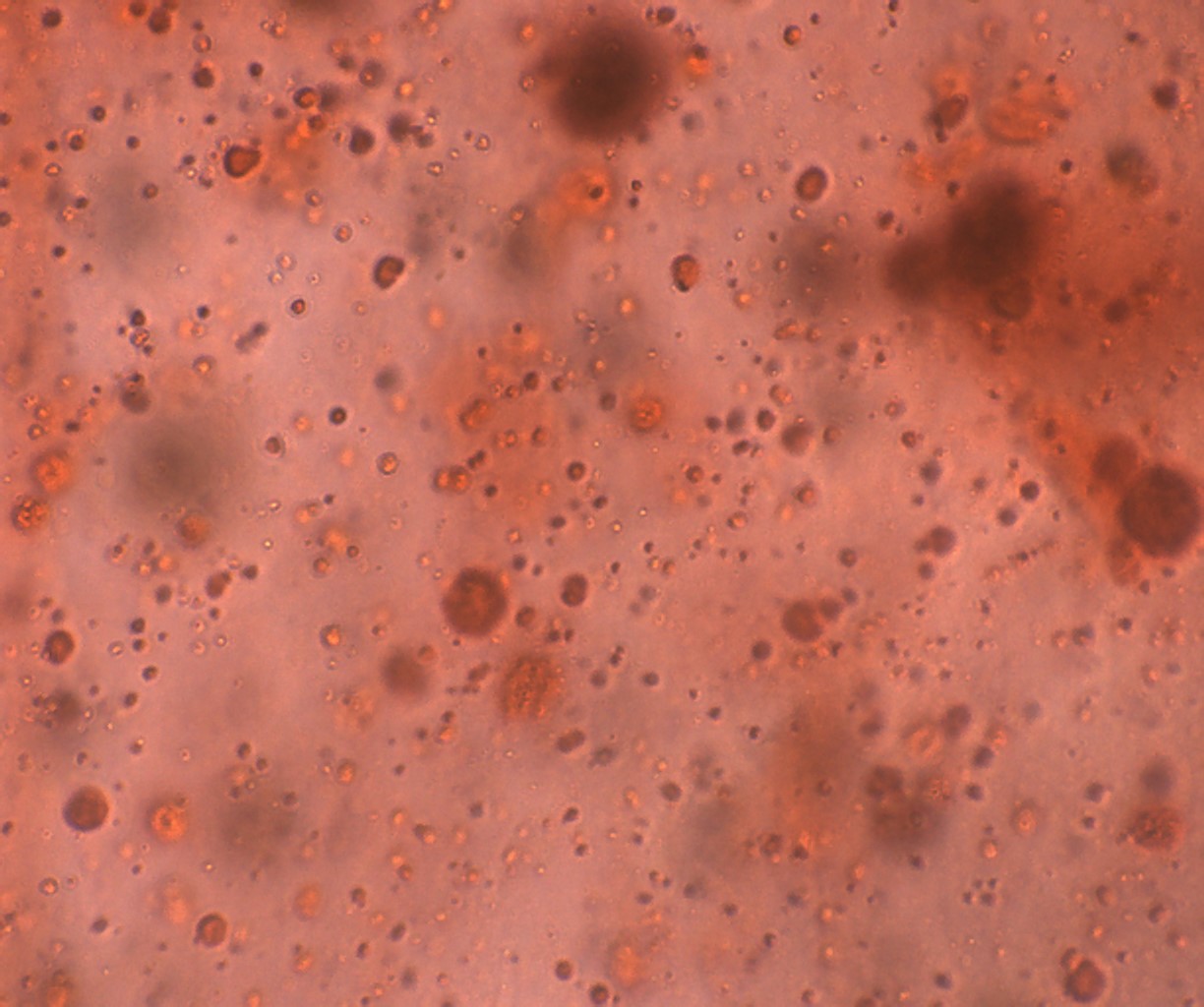

Supplement: Supplementary file 4 — Source Data [file 41467_2025_58761_MOESM4_ESM.zip › Figure 2/biotic day 20 alizarin red 40x.jpeg]

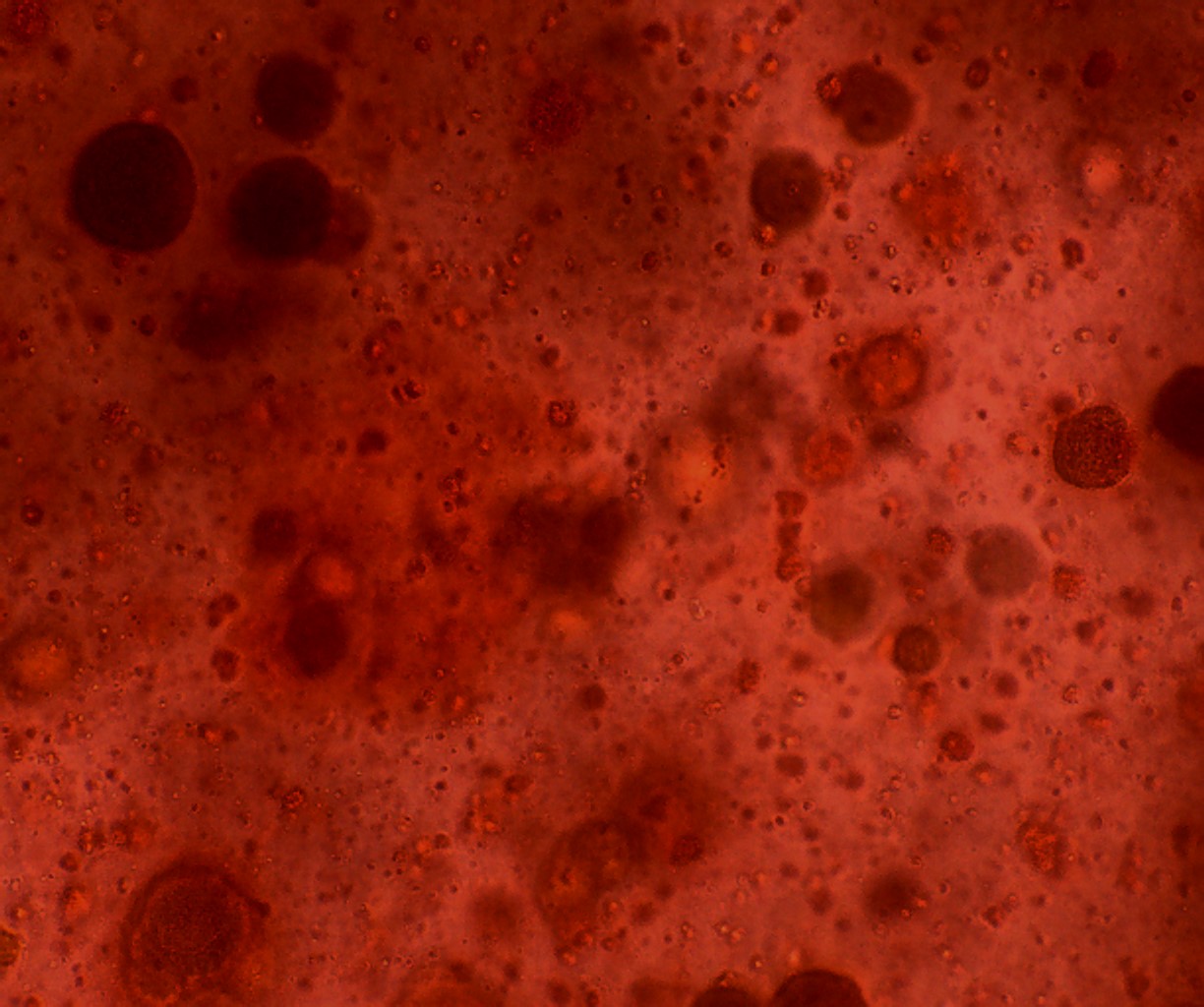

Supplement: Supplementary file 4 — Source Data [file 41467_2025_58761_MOESM4_ESM.zip › Figure 2/biotic day 30 alizarin red 40x.jpeg]
